# Impact of Interferon Lambda 4 Genotype on Interferon‐Stimulated Gene Expression During Direct‐Acting Antiviral Therapy for Hepatitis C

**DOI:** 10.1002/hep.29877

**Published:** 2018-09-15

**Authors:** Narayan Ramamurthy, Emanuele Marchi, M. Azim Ansari, Vincent Pedergnana, Angela Mclean, Emma Hudson, STOP HCV consortium, Rory Bowden, Chris C.A. Spencer, Eleanor Barnes, Paul Klenerman

**Affiliations:** ^1^ Peter Medawar Building for Pathogen Research and Translational Gastroeneterology Unit, Nuffield Department of Medicine University of Oxford Oxford United Kingdom; ^2^ Wellcome Trust Centre for Human Genetics University of Oxford Oxford United Kingdom; ^3^ Oxford Martin School University of Oxford Oxford United Kingdom; ^4^ Department of Zoology University of Oxford Oxford United Kingdom

## Abstract

New directly acting antivirals (DAAs) provide very high cure rates in most patients infected by hepatitis C virus (HCV). However, some patient groups have been relatively harder to treat, including those with cirrhosis or infected with HCV genotype 3. In the recent BOSON trial, genotype 3, patients with cirrhosis receiving a 16‐week course of sofosbuvir and ribavirin had a sustained virological response (SVR) rate of around 50%. In patients with cirrhosis, interferon lambda 4 (*IFNL4*) CC genotype was significantly associated with SVR. This genotype was also associated with a lower interferon‐stimulated gene (ISG) signature in peripheral blood and in liver at baseline. Unexpectedly, patients with the CC genotype showed a dynamic increase in ISG expression between weeks 4 and 16 of DAA therapy, whereas the reverse was true for non‐CC patients. *Conclusion:* These data provide an important dynamic link between host genotype and phenotype in HCV therapy also potentially relevant to naturally acquired infection. (Hepatology 2018; 00:000‐000).

Hepatitis C virus (HCV) infects approximately 180 million people globally. An estimated 20% of patients are able to control the infection spontaneously after exposure—that is, through a combination of innate and adaptive responses.^1^, ^2^ In the remainder, chronic hepatitis leads to HCV‐related cirrhosis in around 15%‐30% of patients and 1%‐4% of patients with cirrhosis develop hepatocellular carcinoma.^3^ The development of treatments for HCV infection has therefore been an important priority in recent years and huge advances have been made.

Interferon (IFN) and ribavirin combination therapy was, for many years, the mainstay of treatment for patients with chronic hepatitis C (CHC).^4^ However, regimes using combined pegylated interferon (Peg‐IFN) and ribavirin therapy result in only modest sustained virological response (SVR) rates and are associated with significant adverse effects. Recently, many new directly acting antiviral (DAA) therapies have been developed, which provide significantly higher cure rates in different patient groups. Such drugs may be used in combination with IFN (e.g., the protease inhibitor, telapravir)^5^ or, most recently, in IFN‐free regimens, using combinations of inhibitors of nonstructural protein (NS) 3, NS5A, and NS5B, with or without ribavirin.^6^

HCV is genetically highly diverse and has evolved into seven major genotypes and multiple subtypes. Response rates to both the IFN‐based and, more recently, the DAA based regimes are genotype dependent. In particular, HCV genotype 3 (one of the most common in the UK) has been associated with lower response rates to several DAA regimens, compared to genotype 1 infection.^7^, ^8^, ^9^ Defining optimal treatment for such patient groups is an issue that was addressed in the recent BOSON trial,^10^ which tested the efficacy of the NS5B (polymerase) inhibitor, sofosbuvir, on 594 patients, mainly genotype 3, with a small subgroup of genotype 2 infections, and including patients with and without cirrhosis and with or without previous treatment. Patients were divided into three groups, given 16 or 24 weeks of sofosbuvir with ribavirin or 12 weeks of sofosbuvir with Peg‐IFN and ribavirin. A key result of the study was that patients with cirrhosis who received 16 weeks of sofosbuvir and ribavirin had the lowest rates of SVR—at 51%, compared to >80% attaining cure in other treatment groups. This prompted us to ask: In such a setting (and in the absence of exogeneous interferon), what is the role of the host immune response and host genetics in defining outcome?

Host genetics play an important role in both the spontaneous control of HCV and response to therapy. In particular, a single‐nucleotide polymorphism (SNP; rs12979860) in the interferon lambda 4 (IFNL4) gene^11^, ^12^, ^13^ has been associated with higher probability of spontaneous clearance of the virus and of SVR following Peg‐IFN and ribavirin combination therapy.^12^, ^14^, ^15^ Those carrying the CC genotype at rs12979860 had a greater than 2‐fold increase in the likelihood of achieving SVR compared to the TT genotype.^12^ The CC genotype was also associated with changes in the dynamics of viral control in genotypes 1 and 3.^12^, ^16^, ^17^, ^18^, ^19^

The mechanism by which the *IFNL4* locus affects hepatitis C outcomes has not been fully elucidated. Although the CC genotype is associated with better immune control and response to therapy, it has also been associated with higher viral load than the non‐CC genotypes.^13^, ^14^, ^15^, ^16^, ^18^ This elevated viral load is associated with a reduced host innate immune response during chronic infection. Hepatic interferon‐stimulated genes (ISGs) are also up‐regulated in CHC patients resistant to antiviral therapy and higher in patients with the non‐CC with *IFNL4* genotype.^20^ These associations may be attributed to modulation of the *IFNL4* gene itself or of linked genes. SNPs in the *IFNL4* region may influence expression, transcription factor binding, or mRNA stability, but overall appear to reduce *IFNL3* gene expression.^21^ The C allele at SNP rs12979860 tags a dinucleotide insertion polymorphism (rs368234815‐TT)^22^ that prevents *IFNL4* expression.^23^ Using human genotyping arrays combined with whole‐genome viral sequencing, we recently reported viral “footprints” of host *IFNL4* genotypes (CC vs. non‐CC at rs12979860). We identified the association of 11 sites in the HCV polyprotein with IFNL4 genotypes, including one (serine vs. nonserine at position 2414 in NS5A) that was also associated with viral load *in vivo* and viral fitness *in vitro*.^24^

Based on these data, we hypothesized that host *IFNL4* genotype might particularly affect viral outcome in patients with cirrhosis treated with the shortest protocol (16 weeks). We aimed to test whether transcriptional changes in the host response were detectable in blood and linked to outcome. We therefore analyzed the host response to HCV by microarray analysis of gene expression in whole blood over time in a subset of patients from the BOSON study. We make a report of a time‐dependent molecular signature detectable in blood associated with *IFNL4* genotypes, which, in itself, is associated with SVR in patients with cirrhosis. We provide a model that explains the paradoxical observations related to HCV and its interaction with the *IFNL4* region.

## Materials and Methods

### STUDY DESIGN, PATIENTS, AND ETHICS

This study was part of the study described earlier.^10^, ^22^ PAXgene and liver biopsy samples came from patients enrolled in the BOSON study. The BOSON study is a phase 3 randomized open‐label trial to determine the efficacy and safety of sofosbuvir with and without Peg‐IFN‐alfa, in treatment‐experienced patients with cirrhosis and HCV genotype 2 infection and treatment‐naïve or ‐experienced patients with HCV genotype 3 infection.^10^ We focused analyses here on patients given DAA therapy without IFN (arms 1 and 2). All patients provided written informed consent before undertaking any study‐related procedures. The study protocol was approved by each institution's review board or ethics committee before study initiation. The study was conducted in accord with the International Conference on Harmonization Good Clinical Practice Guidelines and the Declaration of Helsinki. The study reported here is not a clinical trial, but is based on the analysis of patients from a clinical trial (registration number: 510 NCT01962441).

### MICROARRAY ANALYSIS

Globin‐depleted total RNA (500ng) was analyzed using Illumina Human HT12v4.0 Expression Beadchips according to the manufacturer's instructions (v1.9.0; Illumina, San Diego, CA). Gene expression data were checked and exported using Illumina's GenomeStudio (V2011.1) software.

### HCV VIREMIA, IFNL4 GENOTYPING, RNA EXTRACTION, AND PCR

See the Supporting Information for further details.

### BIOINFORMATIC ANALYSIS

Illumina bead chip output files were processed and analyzed using the R package (https://www.r-project.org),^25^ wherein samples were normalized and gene expression levels calculated to determine statistical significance. Differential gene expression between different groups was assessed by generating relevant contrasts corresponding to the possible comparisons. Statistical testing was performed using the Linear Models for Microarray Analysis (LIMMA) package (https://bioconductor.org/packages/release/bioc/html/limma.html).^26^ Raw *P* values were corrected for multiple testing using the false discovery rate controlling procedure of Benjamini and Hochberg.^27^ Following this correction, adjusted *P* values <0.01 were considered significant. Pathway over‐representation analysis and plotting was conducted using Reactome PA.^28^ We also used the CAMERA package^29^ to assess the enrichment of Reactome pathways. Gene set enrichment analysis (GSEA)^30^ and relative enrichment plots were performed using *HTSanalyzeR* package^31^ from within the R environment.

## Results

### *IFNL4* CC GENOTYPE IS ASSOCIATED WITH SVR IN GENOTYPE 3 PATIENTS WITH CIRRHOSIS UNDERGOING TREATMENT WITH SOFOSBUVIR AND RIBAVIRIN

**Figure 1:**
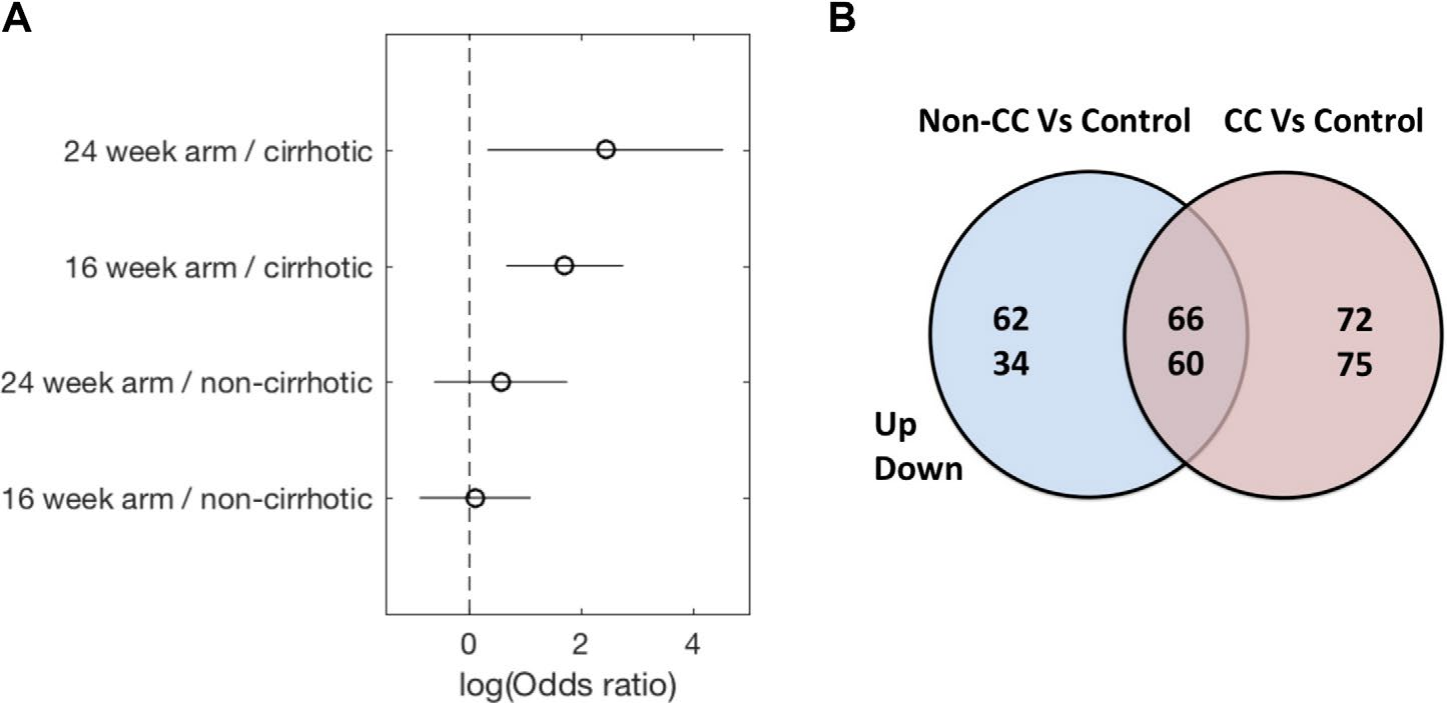
*IFNL4* genotypes and clinical outcomes in the BOSON study. (A) OR graph showing the impact of *IFNL4* genotype on SVR during therapy in chronic HCV patients from the BOSON study (DAA only arms). (B) Summary data from gene expression studies of peripheral blood from patients with cirrhosis taken at baseline. Venn diagram shows differentially regulated genes in IFNL4 CC and non‐CC patients (adjusted *P* value <0.05) when compared to common referenced healthy control group (n = 8).

In the BOSON study, patients were randomised to receive sofosbuvir with ribavirin for 16 or 24 weeks or for 12 weeks with accompanying Peg‐IFN.^10^ To understand the impact of *IFNL4* genotype on attaining SVR, we estimated the effect size (odds ratio; OR) of *IFNL4* CC versus non‐CC on achieving SVR stratified by the cirrhosis status in the two IFN‐free treatment arms. In patients with cirrhosis, those with IFNL4 CC genotype were significantly more likely to achieve SVR (16‐week arm, *P* value = 0.0015; OR = 5.50; CC SVR rate, 76% [26 of 34]; non‐CC SVR rate, 37% [13 of 35]; 24‐week arm, *P* value = 0.009; OR = 11.38; CC SVR rate, 96% [30 of 31]; non‐CC SVR rate, 73% [29 of 40]). However, this was not the case in patients without cirrhosis (16‐week arm: *P* value = 1; OR = 1.1; CC SVR rate, 82% [32 of 39]; non‐CC SVR rate, 81% [62 of 77]; 24‐week arm, *P* value = 0.42; OR = 1.75; CC SVR rate, 90% [36 of 40; non‐CC SVR rate, 84% [67 of 80]; Fig. 1A).

### PATIENTS WITH CIRRHOSIS WITH NON‐CC GENOTYPE HAVE GREATER EXPRESSION OF ISGs IN WHOLE BLOOD

We next addressed whether the host genetic polymorphisms associated with *IFNL4* genotypes (CC vs. non‐CC) were associated with host responses in whole blood using microarray analysis of transcription. We used a healthy control group of 8 individuals as a common reference point and measured up‐ and down‐regulated gene expression in the *IFNL4* CC and non‐CC groups (Fig. 1B). We detected genes, which were exclusively up‐ and down‐regulated in each group (CC or non‐CC) as well as a set of genes expressed in common (threshold for inclusion, *P* < 0.05). Supporting Table S1A,B gives a full list of genes that were up‐regulated in each of the groups of patients.

**Figure 2:**
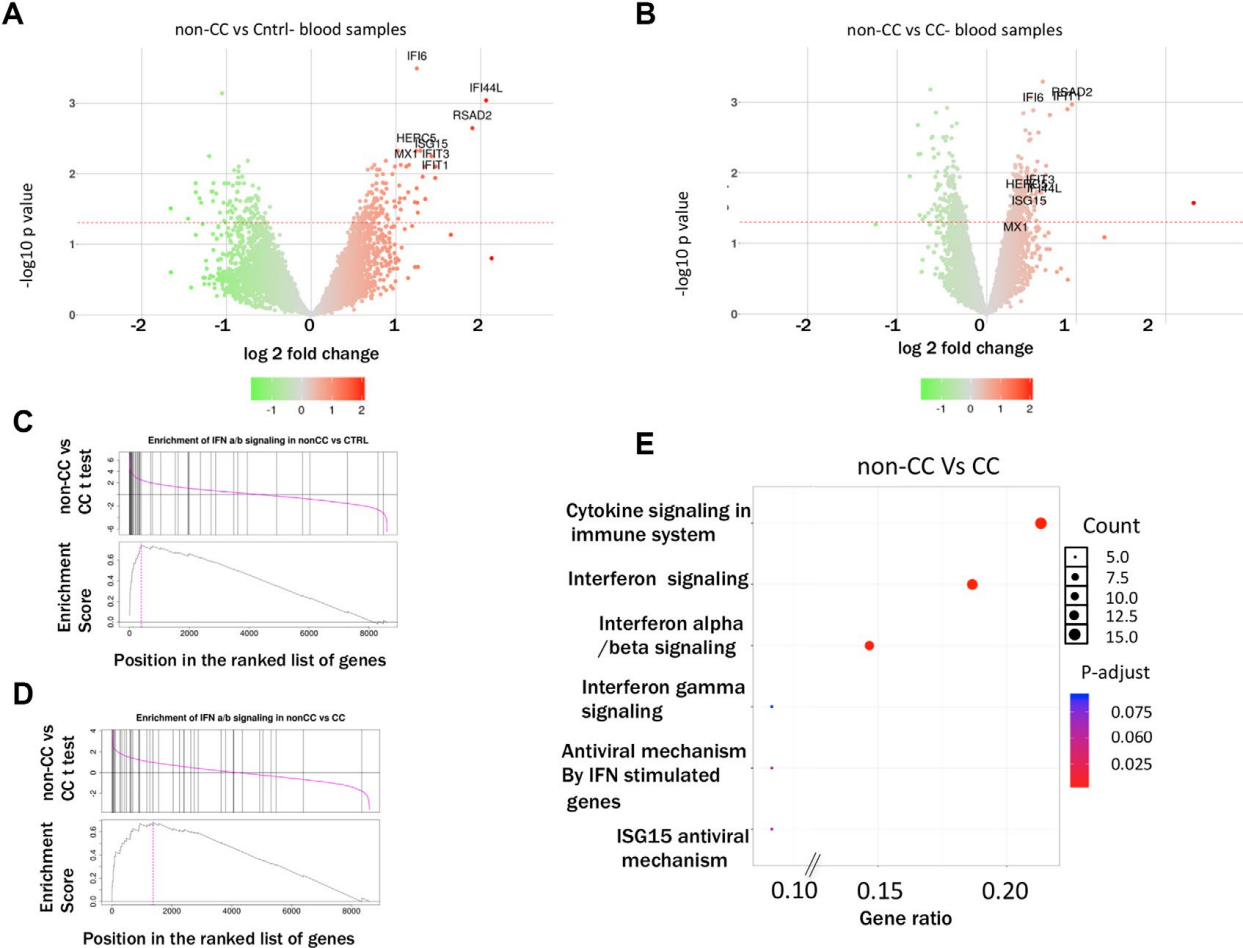
*IFNL4* genotypes and gene expression in peripheral blood during chronic HCV infection. (A,B) Volcano plot showing change in overall gene expression in peripheral blood between non‐CC patients and healthy controls (A) and direct comparison between non‐CC versus CC patients (B) Log2 fold change is plotted along the X‐axis and the *P* value along the y‐axis. The dotted red line shows the threshold of significance (*P* < 0.05). Genes labeled in the figure are those that were validated by real‐time PCR. (C,D) GSEA analysis showing enrichment of *IFNα/β* gene set in non‐CC versus control (C) and a similar comparison of genes comparing non‐CC versus CC patients. (E) *IFNL4* genotypes and pathway analyses during chronic HCV infection: over‐representation analysis of reactome pathways on the list of up‐regulated genes between non‐CC versus CC patient groups. The Y‐axis shows the top six pathways (significance is color coded) X‐axis shows the proportion of gene in each pathway compared to the total list of genes. The size of the circles represent the gene counts in the pathway.

To test (in an unsupervised manner) for over‐representation of certain categories of genes among differentially expressed genes, we checked for statistical enrichment of genes in curated reactome pathways. Among the genes up‐regulated in the non‐CC patients versus healthy controls, we observe a clear and significant enrichment of genes in the IFN α/β and γ signaling pathways (Fig. 2A‐D; Supporting Table S1C,D). The volcano plots (Fig. 2A,B) highlight the general differences in significance and variation in gene expression in non‐CC samples when compared to healthy controls or to CC samples.

The labeled genes in both volcano plots are a selection of transcripts validated by quantitative real‐time PCR (qPCR) known to be representative of an IFN response. Up‐regulation of IFN genes is observable and significant even in CC samples versus healthy controls (data not shown), but is evidently higher in non‐CC samples (Fig. 2E).

Over‐representation of cytokine signaling, IFN signaling, and, specifically, IFN α/β signaling pathways in non‐CC patients was further confirmed by testing enrichments of the reactome gene sets in a direct comparison between non‐CC and CC samples (Fig. 2B); in this “tug‐of‐war” between the two genotypes, expression levels were ranked between the two groups of patients and the analysis clearly showed an over‐representation of cytokine signaling, general IFN signaling and, specifically, IFN α/β signaling. Figure 2E shows the most significantly represented reactome pathways analyzed using Reactome PA,^28^ which takes into account the proportion of genes in each pathway that contribute to the total list of the top ranking genes in the list (*P* < 0.05; lfc > 0). Using an alternate approach to assess the enrichment of reactome pathways (Camera, package),^29^ we performed a gene set enrichment analysis (GSEA). IFN α/β signalling ranks first (*P* value = 1.47e‐09) among a list of categories populated with immunologically relevant pathways (Fig. 2B,D). These data are consistent with previous studies showing enhanced ISG expression in the nonfavorable genotype, although past data have had variable results with respect to genotype 3 and also measurements in peripheral blood.^12^, ^32^

### PATIENTS WITH CIRRHOSIS PATIENTS WITH NON‐CC GENOTYPE HAVE GREATER EXPRESSION OF IFN‐STIMULATED GENES IN THE LIVER

**Figure 3:**
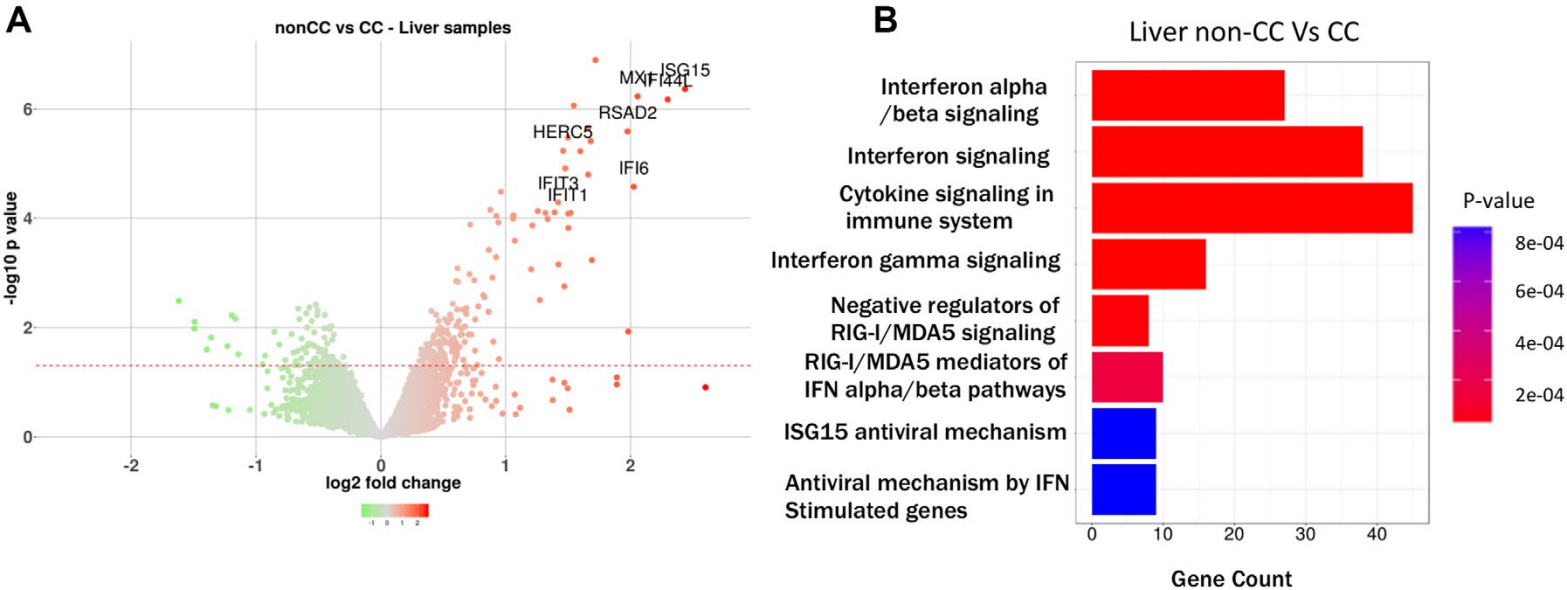
*IFNL4* genotypes and gene expression in liver of HCV patients. (A). Volcano plot showing differentially modulated genes in liver by microarray analysis comparing non‐CC versus CC patients. X‐axis shows log2 fold change and Y‐axis the –log 10 *P* value. The dotted red line shows the threshold of significance (*P* < 0.05). Genes illustrated in the figure are those that were found in the blood in Fig. 1C/D when comparing the non‐CC patients versus healthy control and the non‐CC versus CC groups. (B) Reactome pathways enrichment analysis on differentially up‐regulated list of genes obtained by microarray analysis of liver tissue between non‐CC versus CC patients. The X‐axis shows the gene counts in each pathway and the Y‐axis shows the eight most significant pathways sorted by significance. Abbreviations: MDA5, melanoma differentiation‐associated gene 5; RIG‐I, retinoic acid‐inducible gene I.

**Figure 4:**
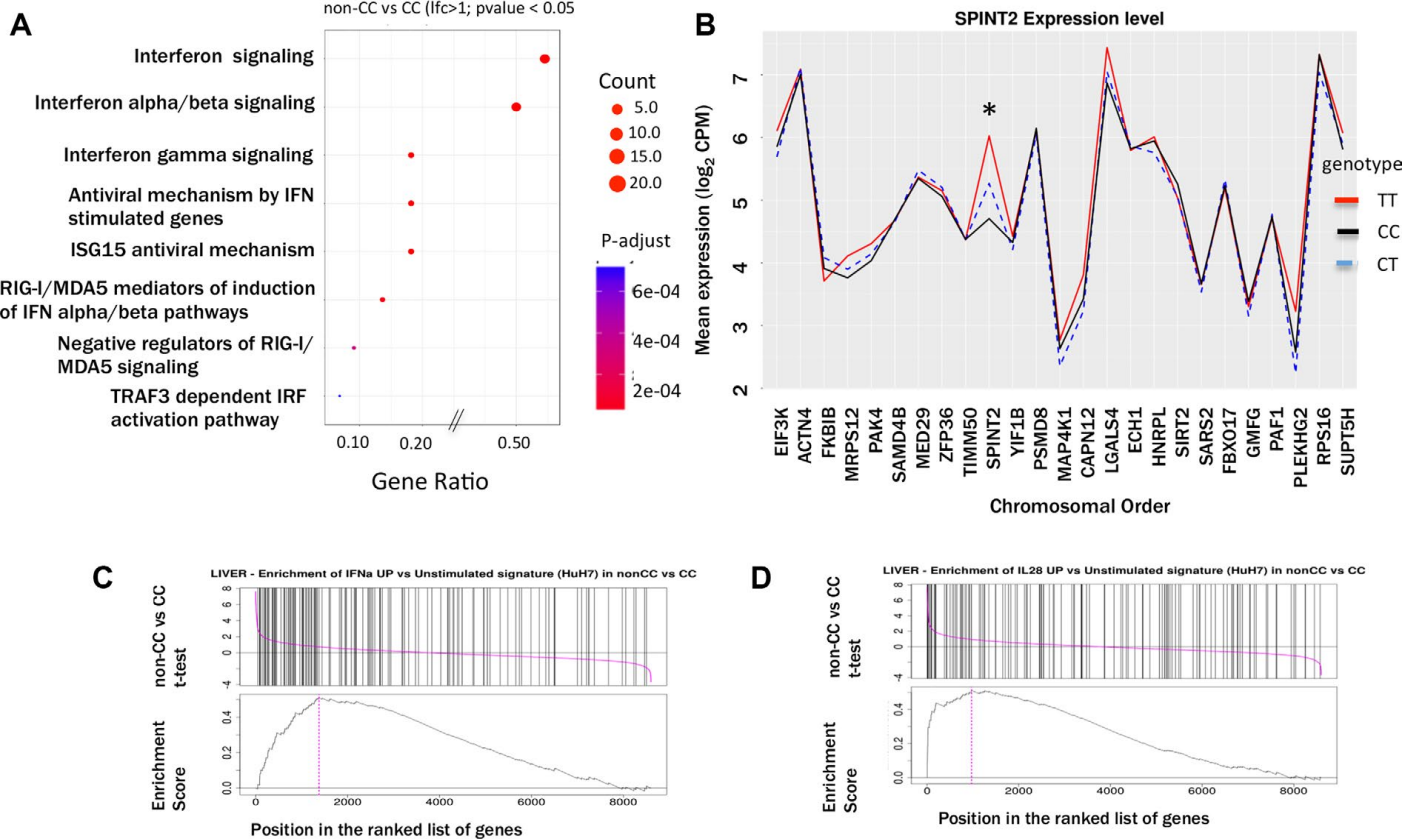
*IFNL4* genotypes and RNAseq analysis in liver of HCV patients. (A) Reactome pathways enrichment analysis on differentially up‐regulated list of genes obtained by RNAseq analysis between non‐CC versus CC patients. Criteria for inclusion were a minimum of 1 lfc (log 2 fold change) in expression and *P* value <0.05. The size of circles reflects the gene count in the pathway, the X‐axis represent the gene ratio (N genes in pathway/n genes in the entire list of genes), and the color of the circles reflects the adjusted *P va*l*ue*. (B) *IFNL4* genotypes and cytoband analysis in liver of HCV patients: expression graph of chromosomally sorted genes in cytoband 19q13.2. Lines across the graph represent average gene expression in each *IFNL4* genotype (TT in red, black dotted lines CT, and black solid line represent expression in CC groups). *IFNL3*/4 gene is not shown because the genes included in the graph are those that have an average gene expression of log2 cpm >2. (C,D) GSEA showing enrichment of *IFNα* and *IFNL* Huh‐7 signature gene (uniquely up‐regulated genes when Huh7 cells are stimulated by IFNα or IFNL3) on non‐CC versus CC gene list in liver. Abbreviations: MDA5, melanoma differentiation‐associated gene 5; RIG‐I, retinoic acid‐inducible gene I.

We also profiled gene expression in a subset of liver biopsy samples (13 biopsies) taken before treatment (baseline) identifying an enrichment of IFN α/β signaling pathways in non‐CC versus CC patients (Fig. 3A,B). RNA sequencing (RNAseq) confirmed enhanced expression of ISGs in the non‐CC population (Fig. 4A).

When genes between non‐CC and CC were compared, pathway analysis (Fig. 3B) showed IFN signalling among the most significantly enriched pathways. To understand whether the enrichment more closely resembles IFNλ3‐driven or IFNα‐driven signaling pathways, we performed a GSEA on these genes and compared them with a preranked list obtained from our previous study on hepatocytes (Huh7) stimulated with IFNα (100 ng/mL) or IFNλ3 (100 ng/mL) for 72 hours (GEO accession: GSE89610).^33^ GSEA analysis showed that genes from both IFNα and IFNλ3 stimulated lists were up‐regulated (Fig. 4C,D and Supporting Fig. S1).

In case the effect of *IFNL4* genotype is mediated by a nearby highly expressed gene, we investigated gene expression in the *IFNL4* region (cytoband 19q13.2) in liver biopsies of patients of CC and non‐CC *IFNL4* genotypes. Expression of *SPINT2* (serine peptidase inhibitor, Kunitz type 2) was most significantly associated: TT carriers had the highest expression followed by CT and then CC genotype patients (Fig. 4B), but the association was observed only in the liver and not in whole blood. No significant association between expression of *IFNL3* or *IFNL4* genes was observed.

### qPCR VALIDATION SHOWS SIGNIFICANT INCREASE IN ISG LEVELS IN NON‐CC PATIENTS

**Figure 5:**
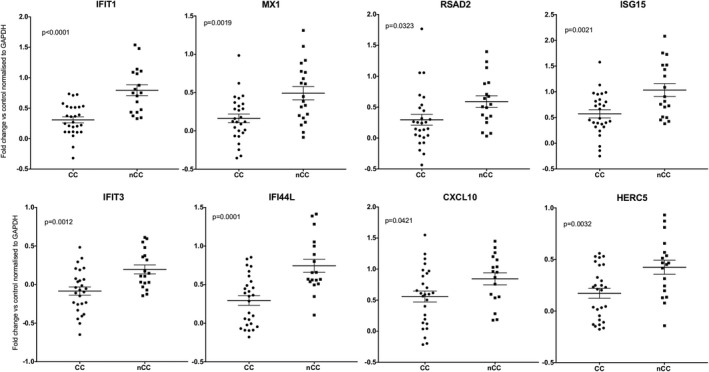
qPCR analysis of peripheral blood gene expression at baseline according to IFNL4 genotype. A set of genes was quantified by PCR from RNA derived from peripheral blood using the relative quantification method with genes normalized against a housekeeping gene (GAPDH). Results from eight ISG gene expressions are shown in a total of 45 patients from the same cohort, of which 27 were CC and 18 patients were non‐CC genotype. Results from qPCR analysis were fed into GraphPad Prism (7.0; GraphPad Software Inc., La Jolla, CA) to generate the graphs. *P* values were generated using the statistical software within Prism using log values of fold changes and SEM is shown. Abbreviation: GAPDH, glyceraldehyde 3‐phosphate dehydrogenase.

We undertook qPCR to validate the enhanced expression of several classical ISGs in non‐CC patients with cirrhosis at baseline (Supporting Table S1B; Fig. 5), expanding the cohort by 17 patients to 45 patient samples in total. In six of eight genes tested, we confirmed a strong association between *IFNL4* status and gene expression levels in peripheral blood. The effect was observed on classical ISGs—*IFIT1*, *MX1*, *ISG15*, *IFIT1*, *IFI44L*, and *HERC5*—where the non‐CC genotype was associated with elevated gene expression, that is, significantly higher than the favorable CC genotype, as well as compared to healthy controls. Of note, these differences in peripheral blood ISG responses were not observed in patients without cirrhosis (Supporting Fig. S2A). Previous groups have shown enhanced expression of ISG in the non‐CC group in the liver,^12^, ^34^, ^35^ but not consistently in blood, and the contrast with our result seems potentially to be attributed to a dilution of the effect when both patients with cirrhosis and patients without cirrhosis are studied.

### DYNAMICS OF ISG EXPRESSION DURING TREATMENT DIFFER BETWEEN *IFNL4* CC AND NON‐CC GENOTYPE PATIENTS

**Figure 6:**
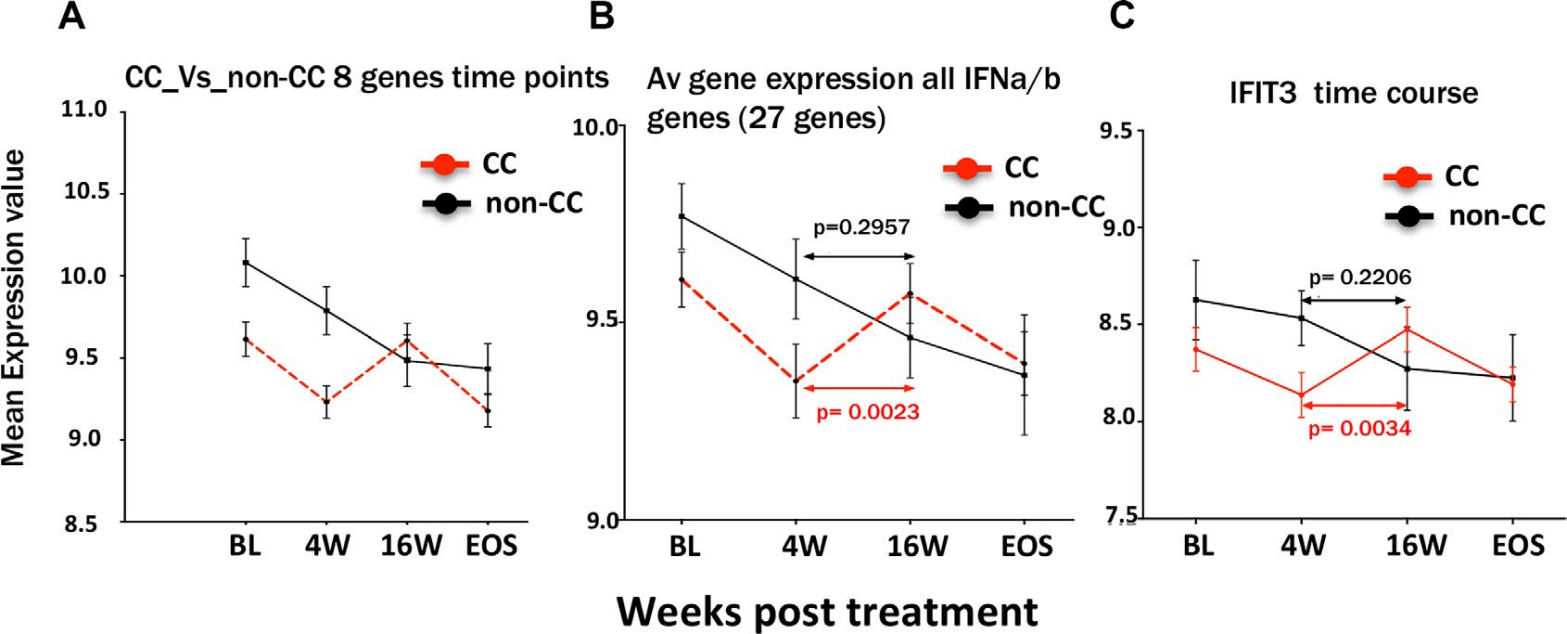
Dynamic changes in peripheral blood gene expression during therapy. (A) Expression of an average of a subset of genes *(RSAD2*, *IFIT1*, *IFIT3*, *IFI44L*, *MX1*, *CXCL10*, *ISG15*, and *HERC5*) over time, that is, BL, 4 weeks, 16 weeks, and at the end of study in CC versus non‐CC patient group. Similar results were obtained when all genes of the *IFNα/β* pathway were analyzed. (B) Gene expression data on the average of 27 genes shown in Table 1 over time as described above. (C) Representative example of gene expression in gene IFIT3 showing the increased expression in the CC patient group at week 16 of treatment compared to week 4. Significance was determined using a paired *t* test. Abbreviations: BL, baseline; EOS, end of study.

We next addressed to what extent gene expression changed over time during therapy, in relation to *IFNL4* genotypes. In both groups (CC and non‐CC), virus was rapidly suppressed and viral RNA PCRs became negative until end of treatment in most cases (Supporting Fig. S2B). We first analyzed a subset of genes (genes analyzed in Fig. 5.) pretherapy, at 4 weeks, 16 weeks, and at the end of study (24 weeks after the end of treatment). Here, we observed an unexpected phenomenon—and a difference between the groups. In the CC group, gene expression was observed to increase toward end of treatment (rising between 4 and 16 weeks). However, the same genes in the non‐CC group do not show the similar increase in expression levels, rather falling in expression over time during therapy (Fig. 6A‐C and Supporting Fig. S3A‐C).

**Figure 7:**
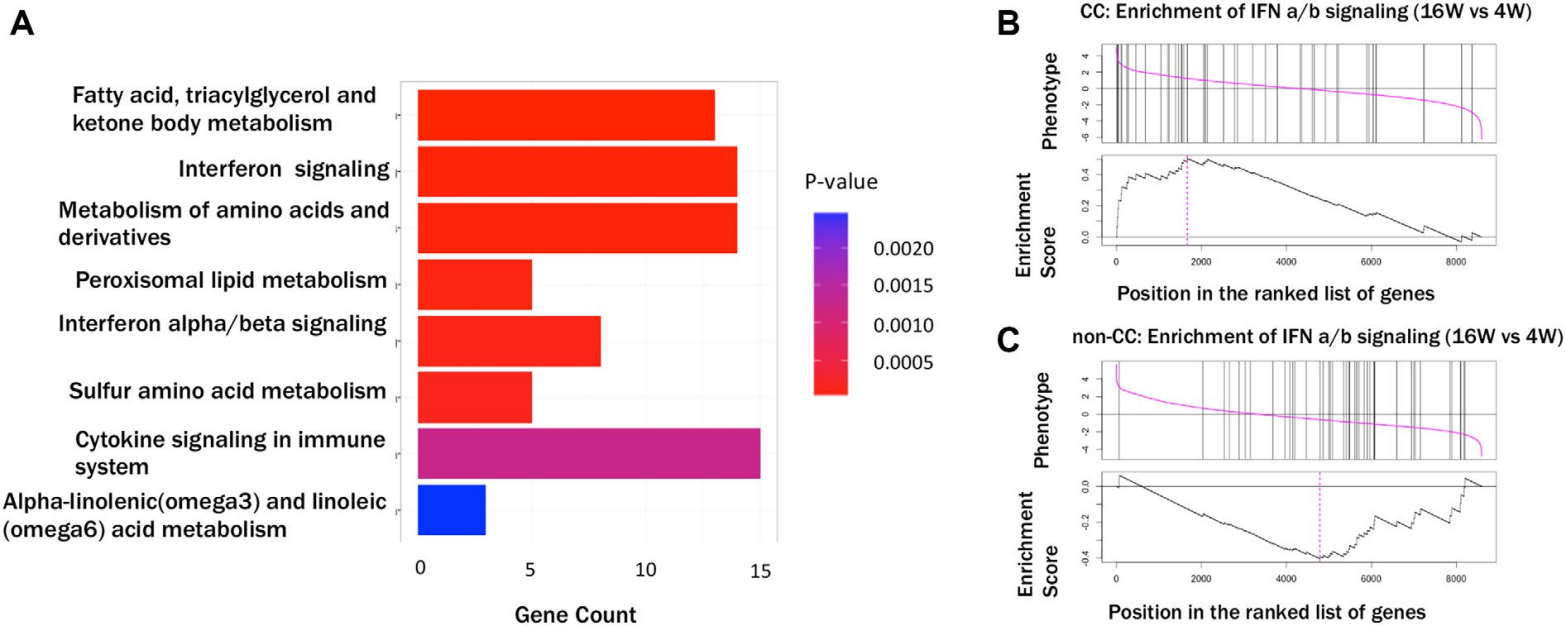
(A) Dynamic changes in reactome pathways during therapy. Over‐representation analysis of reactome pathway genes in CC patients at 16 versus 4 weeks. The up‐regulated list of genes was generated using paired samples at 16 and 4 weeks for each patient and analyzed using paired *t* test. The Y‐axis shows the eight most significant over‐represented reactome pathways (significance color coded) and X‐axis shows gene counts in each pathway. (B,C) Up‐regulation of IFN‐regulated genes according to *IFNL4* genotypes: GSEA analysis showing enrichment of the IFNα/β gene set between 16 versus 4 weeks in CC and non‐CC patient blood samples. The dotted vertical line shows the enrichment “core.” Genes were enriched for the IFNα/β pathway in the CC patient group at 16 weeks. However, in the non‐CC patient group, such an enrichment was not observed.

To understand this increase in gene expression between 4 and 16 weeks in CC patients, and to analyze what sets of genes were up‐regulated, we analyzed the up‐regulation of all genes over this time period in the CC group. Supporting Fig. S4 shows the Venn diagram of differentially regulated genesin IFNL4 CC genotype patients at 4 and 16 weeks of treatment in whole blood when compared to healthy controls, revealing a new set of up‐regulated genes at the later time point. The reactome pathways enrichment analysis on up‐regulated genes between 4 and 16 weeks showed that the genes involved in the IFN α/β gene pathway in the CC group at 16 weeks were among the most represented categories (Fig. 7A; Supporting Table S2A,B).

GSEA analysis of genes of paired samples of the IFN α/β pathway show that patients with the IFNL4 CC genotype show up‐regulation of genes of this host response pathway at 16 weeks compared to 4 weeks (Fig. 7A,B). Importantly, equivalent analysis in the non‐CC group show significant up‐regulation of these genes at 4 weeks rather than at 16 weeks, that is, a down‐regulation over time (Fig. 6B). A contextualized differential expression analysis exclusively on only IFN genes was performed to highlight what genes are the most significant in the context of IFN signaling pathways (Table 1). In this analysis, we also note that there is significant up‐regulation of genes in the intrinsic apoptosis pathway.

**Table 1:** Differential Expression Analysis Performed Exclusively on Genes in the IFNα/β Signaling Pathway (43 Features) in CC Patients Up‐Regulated at 16 Weeks When Compared to Those at 4 Weeks

ID	logFC	AveExpr	t	P.Value	adj.P.Val	B
IFITM3	0.54369485	12.0023006	4.32021235	0.00011602	0.00498869	1.0622434
OASL	0.39797067	8.55867409	3.74314074	0.000629	0.00956154	−0.526625
IFIT1	0.54431309	9.49978316	3.72259827	0.00066708	0.00956154	−0.5815558
STAT1	0.31810156	12.2367207	3.27827781	0.00230924	0.02482437	−1.734864
IFIT2	0.30959571	10.6864439	2.93766556	0.00571849	0.04841688	−2.5658952
MX1	0.30156683	11.3422034	2.87312169	0.00675584	0.04841688	−2.717316
IRF8	−0.2415412	9.74662371	−2.785396	0.00844986	0.05190631	−2.9197644
HLA‐C	0.35011366	10.5071659	2.66580015	0.01140094	0.06128004	−3.1892545
OAS3	0.32389362	8.4497729	2.5733493	0.01430667	0.06835411	−3.3921917
IRF3	−0.2630716	8.94593252	−2.4067598	0.02131318	0.09164667	−3.7453383
IFIT3	0.26489315	9.57162048	2.23364813	0.03176682	0.12417939	−4.0941835
USP18	0.20892888	5.24041669	1.97128898	0.05636246	0.20196548	−4.5848292
ADAR	0.15686838	11.8777284	1.64870926	0.10784976	0.35673383	−5.1195189
ISG15	0.21228863	9.50434984	1.60359579	0.1174832	0.36084126	−5.1878628
XAF1	0.15720552	9.46221489	1.54965909	0.12991208	0.37241463	−5.2674177
PTPN6	−0.1880362	10.8742509	−1.4995268	0.14239785	0.38062752	−5.3392283
IRF5	−0.1719974	6.95892964	−1.4689217	0.15048065	0.38062752	−5.3820457
SOCS3	0.14342655	5.70325015	1.41310533	0.16615553	0.39025422	−5.458117
IRF7	0.14788072	9.83850934	1.39189293	0.17243791	0.39025422	−5.4863376
OAS1	0.13757945	8.02691589	1.32924865	0.1920728	0.39463516	−5.5674377
GBP2	0.13532043	11.5723117	1.32724256	0.1927288	0.39463516	−5.5699791
IFNAR2	0.14983542	9.8565919	1.27620484	0.21000064	0.4104558	−5.6334638
STAT2	0.10353448	10.6768576	1.22034482	0.23021475	0.43040149	−5.7003404
IFI6	0.13818007	10.8491464	1.06586612	0.29353312	0.52591351	−5.8708442
JAK1	0.11415208	8.9418609	1.01099816	0.31871926	0.54819712	−5.9262115
IFITM1	0.10006014	11.9218544	0.96719248	0.33985766	0.56207229	−5.9684321
EGR1	0.09783	7.13441316	0.79499262	0.43179315	0.67667142	−6.1170782
IP6K2	−0.0912588	6.29982658	−0.7797106	0.44062325	0.67667142	−6.1289201
ISG20	−0.0653383	11.5325772	−0.7494629	0.45841531	0.67971925	−6.1517034
HLA‐A	−0.0626801	12.3825849	−0.6908859	0.49403856	0.68575951	−6.1933378
PTPN1	−0.0654316	10.2460777	−0.6761645	0.50322832	0.68575951	−6.2032832
IRF4	0.07786415	6.50909709	0.64195778	0.52494027	0.68575951	−6.2255858
HLA‐G	0.06172342	10.2813706	0.63987139	0.52628055	0.68575951	−6.2269095
PSMB8	0.04403661	10.5311175	0.44826974	0.65662689	0.83043989	−6.3304112
IFI27	−0.0774203	10.1136656	−0.4171174	0.67905287	0.83426495	−6.3438388
OAS2	−0.0359996	9.79019653	−0.3883369	0.70003847	0.83615706	−6.3553932
IFI35	0.02987224	9.50135665	0.31427078	0.75511728	0.87756873	−6.3813588
MX2	−0.0350638	10.1767308	−0.2755001	0.78449668	0.88771992	−6.39278
SOCS1	0.02139765	6.50760493	0.20060399	0.84212968	0.92850196	−6.4106056
IRF6	−0.0125211	4.88767786	−0.1426073	0.88739013	0.93684233	−6.420564
IRF2	−0.0163081	6.95712133	−0.1351153	0.89326826	0.93684233	−6.4216053
HLA‐F	−0.0030076	10.4839197	−0.0284221	0.97748143	0.98520595	−6.4303397
IRF1	−0.0030119	11.5593311	−0.018671	0.98520595	0.98520595	−6.4305696

The gene list was generated by *limma* and genes are sorted by t‐statistic.

### Discussion

In this study, we analyzed the host response in blood and liver of patients suffering from chronic HCV genotype 3 infection, recruited in the BOSON trial. In these analyses, we selected the group that had the lowest response rate to the DAA therapy (sofosbuvir and ribavirin), with an SVR of around 50%. It is in this group where a host response might have the largest demonstrable impact.

Previous analyses of this study have revealed that both *IFNL4* and cirrhosis have an impact on outcome in a multivariate model, together with male sex and treatment arm, baseline viral load, and baseline alanine aminotransferase.^10^ In the current study, the interaction between cirrhosis and *IFNL4* status is clearly demonstrated. Interestingly, in the absence of cirrhosis, we could not detect a significant impact of IFNL4 genotypes in the analyses performed (DAA therapy arms only). This feature suggests that a further host factor modifies the impact of a genetic risk. One simple explanation is that the effect of the *IFNL4* locus gene variant is only detectable in settings where the treatment efficacy is low. However, the SVR at 12 weeks (SVR12) rate in the 24‐week DAA only arm among patients with cirrhosis (where a clear *IFNL4* effect was observed) was 79% in this study, comparable to the 80% response rate in the 16‐week arm among patients without cirrhosis (where no *IFNL4* gene effect was observed). Furthermore, previous multivariate analyses did not reveal an independent effect of cirrhosis in the 24 week treatment group.^10^ An alternative explanation therefore is that there is a true interaction between these biological variables, and the protective mechanisms induced by a favorable genotype have additional relevance in viral control in cirrhosis.

Although we did not address the mechanism behind this interaction, we speculate that an additional critical role for host responses is observed in cirrhosis because of the disrupted microanatomy and immunological milieu of the liver. For example, functionality of the dominant MAIT cell population in the liver is impaired in cirrhosis.^36^ This cell type can secrete potentially potent IFNγ following stimulation by IFNα.^37^ Rescue of responses in these resident populations through a boost of local IFNα may lead to amplified intrahepatic effectors crucial in clearance of residual infection. Alternatively, attributed to disruption of tissue continuity, higher local concentrations of IFNs are required for clearance of all infected hepatocyte niches.

We were able to clearly demonstrate differences in whole‐blood ISG expression between *IFNL4* CC and non‐CC patients. The increase in baseline expression of genes in the *IFNL4* non‐CC genotype in chronic HCV patients observed (Supporting Fig. S1A,B) has been previously shown^20^, ^38^ with increase in ISG at baseline in non‐SVR, but this difference in ISG expression was significantly detected in liver and not in whole‐blood RNA expression.^20^, ^38^, ^39^ In a study of HCV genotype 1 patients with similar DAA for 24 weeks,^40^ liver biopsies collected pretreatment and at end of treatment showed down‐regulation of ISGs, although (consistent with our data) those with an SVR showed higher end‐of‐treatment intrahepatic ISGs than those without. There are a number of differences in our study—first of which is the genotype (focused on genotype 3). Most interestingly, however, we focused additionally on patients with cirrhosis. When we analyzed specific genes by qPCR in patients without cirrhosis at baseline, we observed no significant difference from controls or between *IFNL4* CC and non‐CC groups. These data suggest that the presence of cirrhosis in CHC patients exposes a systemic ISG signal, which is effectively contained within the liver in patients with milder fibrosis. This may reflect the magnitude of the response or potentially a difference in the physiology of the cirrhotic liver, which primes or amplifies systemic responses. It should be noted that the patients recruited to the BOSON study had stable, well‐compensated cirrhosis.

The findings above represent an extrapolation of data that is already available regarding the impact of host genotype on response to HCV.^41^ However, the most unexpected and challenging findings came in the analyses of the dynamic changes in responses on therapy. In non‐CC patients, during DAA treatment, levels of ISGs stabilized and did not show any dynamic oscillation. In contrast, numerous gene sets were activated during DAA therapy in the CC group. Comparison of RNA of whole blood in paired samples from patients of the *IFNL4* CC groups at 4 and 16 weeks, using reactome analysis of the genes up‐regulated over this period, shows up‐regulation of IFN α/β and IFNγ pathways, among others, significantly represented in matching samples (Supporting Table S2A).

How may such transcriptional changes impact on the outcome of therapy in a DAA‐only regimen? Because of the very large impact of *IFNL4* genotype on outcome, in this relatively small study, the same changes we have described as associated with genotype are associated with SVR12. Interestingly, on examining the groups closely, 2 patients who had non‐CC genotype and attained SVR show a gene expression at weeks 4 and 16 similar to that of patients from the CC group (CC SVR vs. non‐CC SVR, data not shown). If this transcriptional response is responsible for clearance, as opposed to being correlated with a further biological effector, then there are a number of possibilities, based on the effects of IFNα, IFNλ, and also IFNγ. IFNα itself could drive the elimination of virus through up‐regulation of a number of effector molecules^42^—especially viruses which have not adapted *in vivo*. There is considerable overlap between the ISGs induced in hepatocytes by IFNα and IFNL3^43^ whereas the expression of IFNL4 itself appears to be highly suppressed by the host.^44^ As we have recently identified,^22^ mutations in the *IFNL4* locus are strongly associated with viral adaptations. These mutations are also associated with viral load, and the likely explanation is that they could provide resistance to the impact of endogeneous ISGs, although the exact mechanism requires further exploration. Previous studies have also indicated that elimination of HCV may be orchestrated by *IFNγ*‐driven expression.^45^

Modeling of the dynamics of HCV under therapy indicates that destruction of persistently infected cells could play a role in long‐term clearance.^46^ Our findings may be relevant to this idea given that activation of ISGs can be linked to induction of cell death mechanisms.^47^ This is evident from our own data, which indicate cell death pathway up‐regulation both using *in vitro* IFNα and in the DAA trial (Supporting Table S2A). The up‐regulation of relevant gene pathways and cell death toward the end of therapy would provide a plausible explanation for the impact of *IFNL4* polymorphisms in this context. A similar effect has been demonstrated with host IL28B genetic differences associated with differential effects on beta‐phase decay in viral load; the more rapid decay is thought to reflect enhanced loss of infected hepatocytes in the CC genotype.^48^

The most important aspect in our view of this finding is the paradoxical increase in ISGs observed during viral suppression. One parsimonious explanation for this finding is that of reduction in an inhibitory pathway suppressing responses to viral RNA in the CC group. A simple model is as follows: With the favorable *IFNL4* genotype, *IFNα* and *IFNL3* are induced early (without *IFNL4*). This is either associated with viral clearance or, if not, rapidly damped such that in chronic infection, low levels of IFN gene expression are observed, with low levels of ISGs and higher viral loads. In the non‐CC genotype, IFNs are induced, and if the virus is not cleared, their expression is not damped, leading to high levels of ISGs and a modest reduction in viral loads. Upon DAA‐induced virus control in the favorable genotype, IFNL3 and/or IFNα induction are no longer damped and can be reinduced in response to residual levels of viral RNA. In the non‐CC genotype, IFN levels simply decline in response to viral suppression. Thus, the critical difference between genotypes under this model is not the specific activity of the molecules, but the sensitivity of the regulatory networks to damping. This model, that genetic polymorphism drives dynamic changes in regulatory networks following an intervention, could provide a paradigm relevant to other settings where attempts to link genotype and phenotype are complex. A model for the hypothesis is shown in Supporting Fig. S5.

Finally, because *IFNL3* and *IFNL4* transcripts were barely detected in both liver and whole blood in these patients, we investigated differential transcriptional activity in nearby genes in the same cyto‐genetic band (19q13.2 comprising 86 genes in 6 Mbp region). We did not detect *IFNL3/4* gene expression in liver (microarray and RNAseq data) or blood samples, nor immediately surrounding genes. However, other genes in the cytogenetic band showed high level of expression, and above all a significant difference was observed between the genotypes. Of note, *SPINT2* was more expressed in the non‐CC patients. Further analysis revealed highest expression in the TT genotype, dropping in the CT genotype and then the CC genotype (Fig. 4E). It is also of note that SPINT2 is found to be associated with hepatocarcinoma,^49^ and exon sequencing analysis in HCV genotype 1b patients showed an SNP (rs3745948) in SPINT2 to be one of the 39 SNPs significantly associated with spontaneous clearance.^50^

Overall, our data indicate a clear impact of *IFNL4* genotype on clinical outcome in patients with cirrhosis treated with a suboptimal DAA regimen, together with a clear association between IFNL4 genotypes and gene expression. These data suggest that a significant role remains for host immunity in such patients and also suggest that the detected changes in gene expression linked to the IFNL4 region drive favorable outcomes. In a clinical setting where liver biopsy is rarely performed, peripheral blood gene expression studies can provide significant signals to link genotype, gene expression, and clinical response. However, further work is required to understand the physiological basis for the powerful impact of cirrhosis on outcome in relation to genotype.

Author names in bold designate shared co‐first authorship.

**Abbreviations**
DAA: direct‐acting antiviral
GSEA: gene set enrichment analysis
HCV: hepatitis C virus
IFN: interferon
IFNL4: interferon lambda 4
ISG: interferon‐stimulated gene
NS: nonstructural protein
OR: odds ratio
Peg‐IFN: pegylated interferon
RNAseq: RNA sequencing
SNP: single‐nucleotide polymorphism
SVR: sustained virological response
