# Supplementary material for: Impact of Interferon Lambda 4 Genotype on Interferon‐Stimulated Gene Expression During Direct‐Acting Antiviral Therapy for Hepatitis C
Source: Hepatology. 2018 Sep 15;68(3):859–71. doi: 10.1002/hep.29877 (PMC6207923; doi:10.1002/hep.29877)
Supplement: Supplementary file 1 [file HEP-68-859-s001.pdf]

Supporting information

Hepatitis C viremia: Plasma HCV RNA was analyzed using the Roche Cobas TaqMan HCV RNA CAP/ CTM Test, v2.0 for use with the High Pure System (Roche Molecular Systems, Inc., Branchburg, NJ), which has a lower limit of quantification (LLOQ) of 15 IU/mL (1).

HCV genotyping was based on whole genome sequences compared to a set of reference sequences annotated by ICTV(2)

IFNL4 genotyping

To perform the genotyping analysis, genomic DNA was extracted from PBMC collected in 0.5M EDTA. Genotyping was performed using Affymetrix UK Biobank array. *IFNL4* SNP rs12979860 is directly typed in this array.

RNA extraction:

Total RNA from blood collected in Paxgene tubes were extracted using the Paxgene Blood RNA kit (Qiagen 762174)following manufacturers instruction. Briefly, the frozen blood collection tubes were thawed at RT and let to further incubate for 2 hrs, and centrifuged for 10 min 4000 xg.

The supernatant was discarded and the washed with water, centrifuged and the pellet was treated with proteinase K incubated at 55°C for 10 mins on a shaker heat block. The resulting lysate was then passed through the Shredder spin column, topped up with 0.5 vol of 100% ethanol. This mix was then passed through the RNA spin column washed once and the column was incubated with DNase, washed further and finally the RNA was isolated using 100 ul of elution buffer. The eluate RNA was then incubated at 65°C incubator for 5 mins and immediately chilled on ice. The resulting RNA was then assessed for yield and quality using a Nanodrop and frozen at -20°C or -70°C.

Globin clear:

As globins are predominant in the RNA sample, we used the Invitrogen globin clear kit (Ambion AM1980) to improve the global expression screening of

samples prior to analysis by microarray analysis following manufacturers instruction.

Briefly, 1-10ug total RNA was hybridised with biotinylated Globin capture oligo beads for 15 mins . This step tags globin mRNA in the sample with biotinylated beads. The tagged RNA is then washed and further incubated with Streptavidin magnetic beads at 50°C for 15 mins. The streptavidin-biotin tagged RNA was then washed with buffer and placed on a magnetic stand that separates the labelled Globin RNA from the rest of the RNA which is collected by aspiration, quantified and stored at -80°C until ready for microarray analysis.

Real time PCR validation:

Reverse Transcription: Approximately 500ng of total RNA from blood was reverse transcribed using the Superscript reverse transcriptase III using OligodT and random hexamer primers. (Invitrogen). The resulting cDNA was used for qPCR as described below.

qPCR: 5ul of a 1/20th dilution of the cDNA was used as a template for qPCR on the Roche light cycler 480 to detect expression of selected ISGs: *IFIT1, MX1, RSAD2, ISG15, IFIT3, IFI44L* and *HERC5*. Primers were synthesized as suggested by the universal probe library assay design centre. Primer and probe sequences are provided in Table below. Relative quantification of genes were performed (48) using the ΔΔCT method normalizing to GAPDH or beta-actin as reference genes and further normalized to control RNA from healthy individuals. Values were analysed using GraphPad Prism (version 7) and statistical analysis on data were generated by t-test analysis within Prism software.

Supplimentary table 1

Table showing Primer and Probes used in the study:

Gene	Primer Sequence	Probe Number	NCBI Accession No.
IFIT1	For- 5’-AGAACGGCTGCCTAATTTACAG- 3’<br>Rev-5’-GCTCCAGACTATCCTTGACCTG-3’	9	NM_001548
IFIT3	For- 5’-TTTTCGGAACAGCAGAGACA-3’<br>Rev-5’-ATGGCATTTCAGCTGTGGA-3’	80	NM_001549
RSAD2	For- 5’-TGCTTTTGCTTAAGGAAGCTG-3’<br>Rev-5’-AGGTATTCTCCCCGGTCTTG-3’	39	NM_080657
ISG15	For- 5’-GCGAACTCATCTTTGCCAGTA-3’<br>Rev-5’-CCAGCATCTTCACCGTCAG-3’	23	NM_005101
MX1	For- 5’-TTCAGCACCTGATGGCCTA-3’<br>Rev-5’-AAAGGGATGTGGCTGGAGAT-3’	79	BC032602
IFI44L	For- 5’-TGACACTATGGGGCTAGATGG-3’<br>Rev-5’-TTGGTTTACGGGAATTAAACTGAT-3’	15	NM_006820
HERC5	For- 5’-CACAGAATGAGCTAAGACCCTGT-3’<br>Rev-5’-AGAAACATAGGCAAGTGTGTGC-3’	83	AY337518

1. Foster GR, Pianko S, Brown A, Forton D, Nahass RG, George J, et al. Efficacy of Sofosbuvir Plus Ribavirin with or Without Peginterferon-Alfa in Patients with Hepatitis C Virus Genotype 3 Infection and Treatment-Experienced Patients with Cirrhosis and Hepatitis C Virus Genotype 2 Infection. Gastroenterology [Internet]. 2015;149:1462–1470. Available from: http://dx.doi.org/10.1053/j.gastro.2015.07.043

2. Smith DB, Bukh J, Kuiken C, Muerhoff AS, Rice CM, Stapleton JT, et al. Expanded classification of hepatitis C virus into 7 genotypes and 67 subtypes: updated criteria and genotype assignment web resource. Hepatology [Internet]. 2014 [cited 2017 Jun 9];59:318–27. Available from: http://www.ncbi.nlm.nih.gov/pubmed/24115039

Suppl.. fig 1: Venn diagram showing differentially expressed genes (adjusted P value <0.05) of Huh-7 cells when stimulated with IFNα and IFNL3 (the numbers above represent up-regulated genes and the one below represent down-regulated genes. In red are uniquely up-regulated genes in each group.

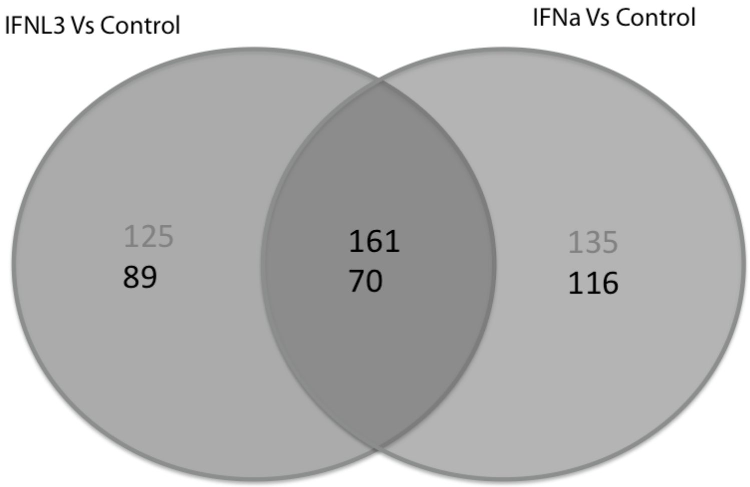

Suppl.. fig 2a: qPCR analysis comparing CC Vs non-CC in non-cirrhotic patients. Analysis done on GraphPad Prism and t-test performed within Prism.

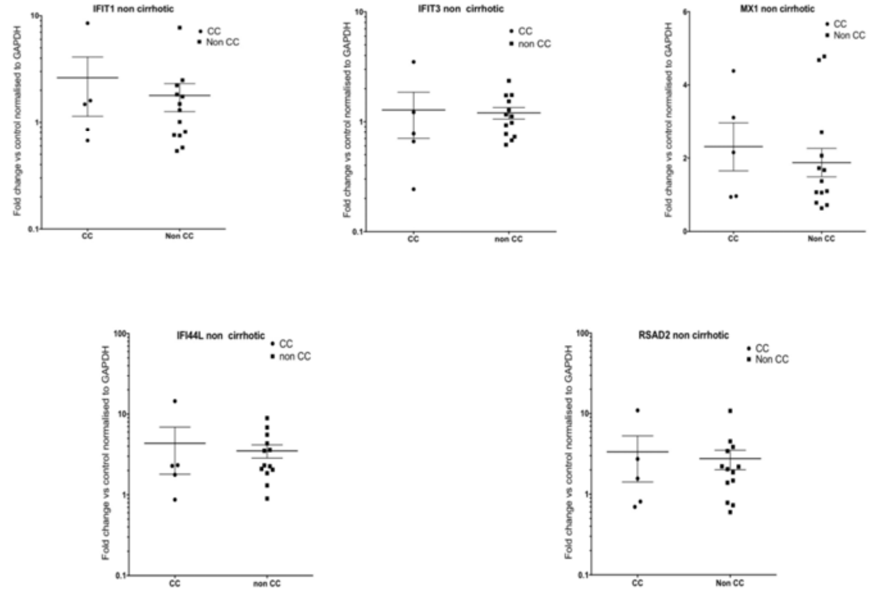

Suppl.. fig 2b: Viral load data in patients during course of therapy

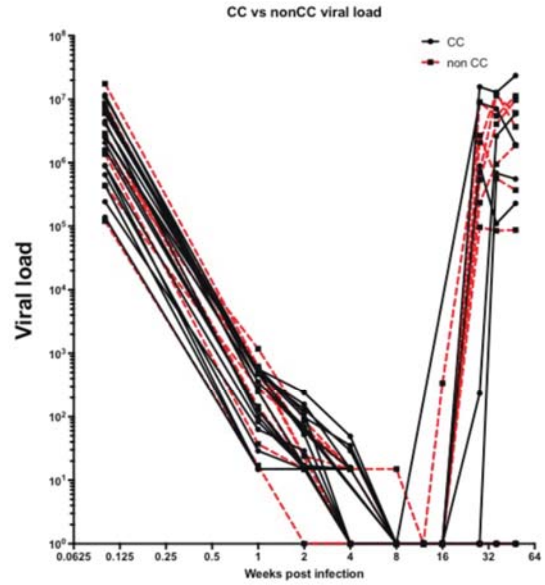

Suppl Fig 3a showing mean values of gene expression from all patients

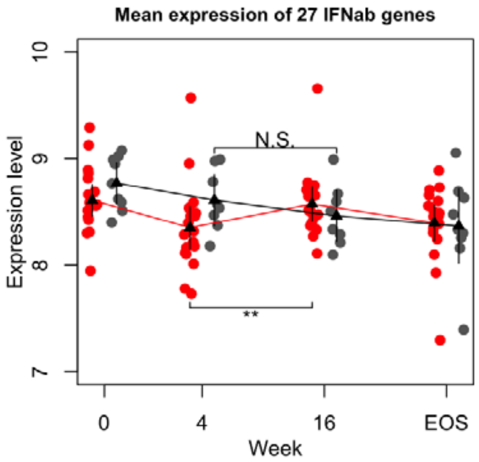

Suppl fig 3b and 3c. Showing individual values of gene expression analysed in the study

Suppl fig 3b

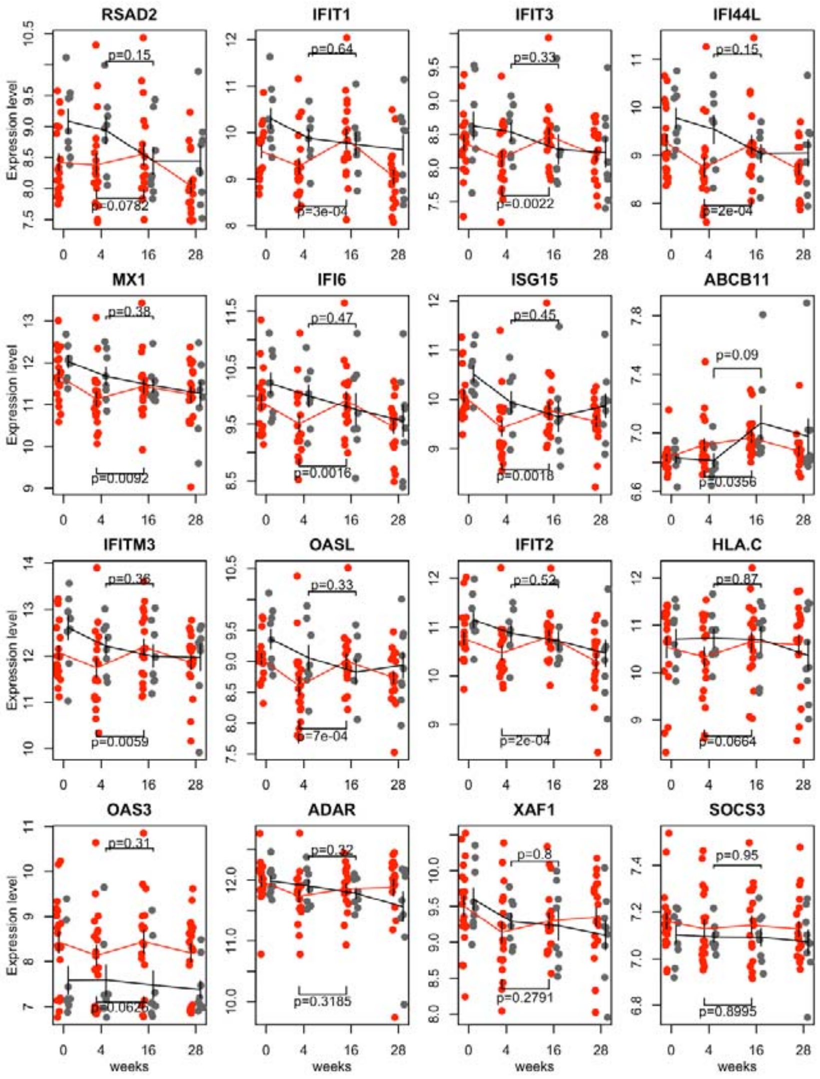

Suppl fig 3C

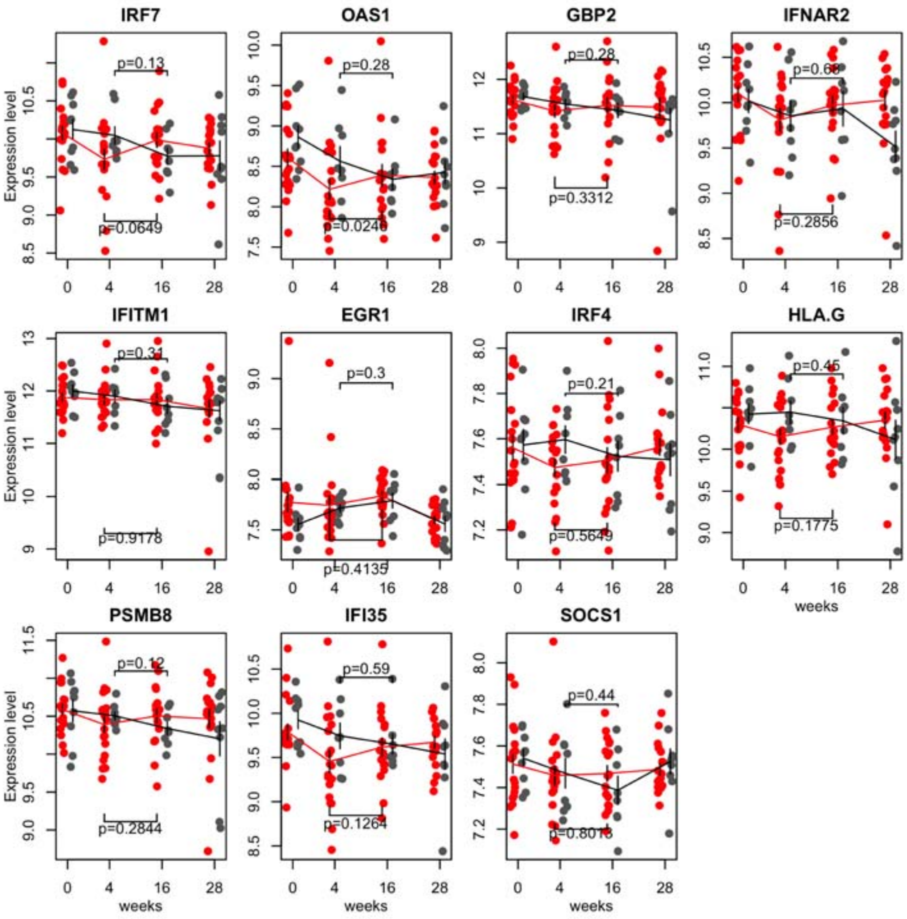
IRF7
OAS1
GBP2
IFNAR2
IFITM1
EGR1
IRF4
HLA.G
PSMB8
IFI35
SOCS1
Expression level
weeks
p=0.13
p=0.0649
p=0.28
p=0.0240
p=0.28
p=0.3312
p=0.68
p=0.2856
p=0.31
p=0.9178
p=0.3
p=0.4135
p=0.21
p=0.5649
p=0.45
p=0.1775
p=0.12
p=0.2844
p=0.59
p=0.1264
p=0.44
p=0.8018

Suppl Fig 4. Venn diagram showing differentially regulated genes in CC patients at 4 weeks and 16 weeks of treatment in whole blood when compared to a common Control (Healthy volunteers). Paired t test with adjusted p value <0.05 performed in LIMMA.

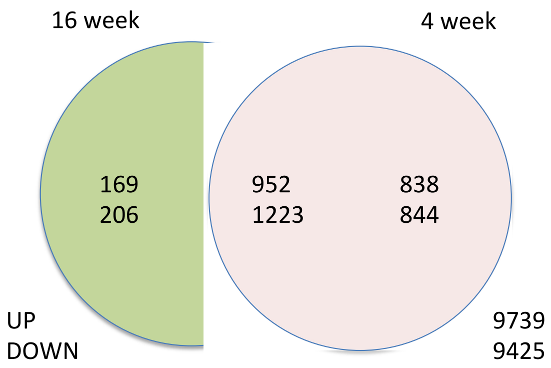

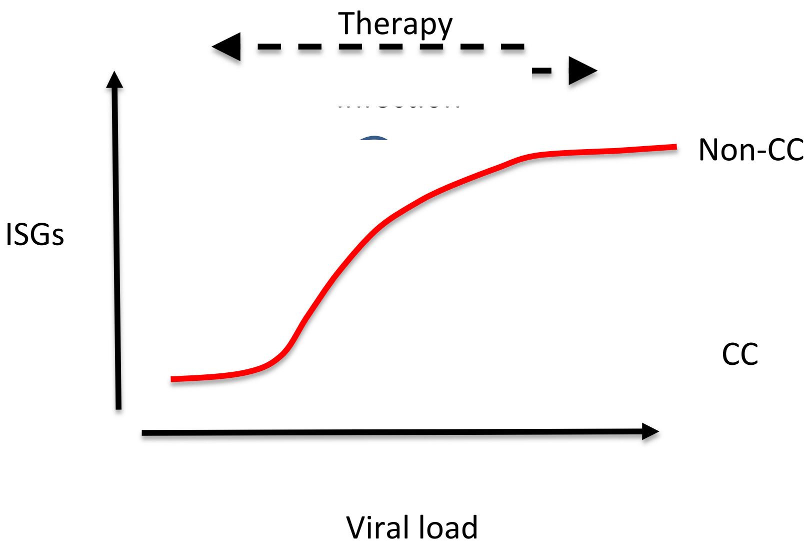

Supplementary Figure 5: Model for dynamic changes in ISGs in relation to host genotype.

Our data could be explained by a model whereby the CC genotype undergoes "damping" at higher viral loads and the non-CC genotype does not. As infection progresses, if it is not cleared, a steady state would be reached where, as seen, ISG levels are lower in the CC genotype (figure is traversed left to right). As virus is cleared by DAA therapy, the ISG response is "undamped" and a peak is seen as the figure is traversed right to left. The shape of the curves could be determined by the differential cross-regulation of Type III and Type I interferons driven by the underlying genotype.

Suppl.. Table 1a: List of genes differentially regulated at baseline between CC vs Control sorted by p value change in ascending order. Adjusted P value was generated using moderated t-test in limma package.

SYMBOL	logFC	adj.P.Val	SYMBOL	logFC	adj.P.Val	SYMBOL	logFC	adj.P.Val
REC8	0.797284	0.003587	RPS15A	0.808451	0.042143	TIMP2	-0.73550	0.03262
PAQR8	0.706403	0.004997	N6AMT1	0.665838	0.042143	HS1BP3	-0.66096	0.03263
GVIN1	0.668675	0.007192	JMJD7-PLA2G4B	0.557000	0.042143	NAT8B	-0.87671	0.03263
LOC100188949	0.618743	0.011234	SMYD2	0.506118	0.042143	SDCBP	-0.66027	0.03263
RPL15	0.647320	0.012077	EIF4A1	0.643816	0.042143	SF3B1	-0.64013	0.03263
UAP1L1	0.629079	0.013662	UBA7	0.469454	0.042143	SUV420H1	-0.48336	0.03263
BX114974	0.767773	0.014100	TRA2A	0.580336	0.042246	PGAM1	-0.57979	0.03377
BX116720	0.691165	0.017487	MECP2	0.494383	0.042439	LOC100129034	-0.56986	0.03377
CDKN2D	0.769569	0.025257	AMMECR1	0.589144	0.042960	TUBA1B	-0.55758	0.03377
CD72	0.868061	0.025966	PHF5A	0.549313	0.043477	RXRA	-0.69176	0.03458
SCARNA21	0.692921	0.025966	RPL23AP64	0.433984	0.044222	ABCC3	-0.88289	0.03463
HBD	1.783446	0.026387	AP1G2	0.487512	0.044961	SRPK2	-0.57163	0.03521
KIAA0454	0.614869	0.027028	BG436024	0.468369	0.044961	VRK3	-0.57762	0.03521
BIRC3	0.694498	0.027381	IRF5	1.059388	0.044961	PLEKHB2	-0.67577	0.03711
MPRIP	0.612295	0.028413	PARP14	0.568358	0.046340	PTGDS	-1.11830	0.03711
SCAND2	0.628130	0.028413	RPS15A	0.825848	0.046366	CTDSPL	-0.81151	0.03788
METTL17	0.511178	0.028467	ROMO1	0.572607	0.047895	POTEKP	-0.58707	0.03788
RELL2	0.528428	0.028467	LOC100505715	0.622322	0.047895	DNAJB6	-0.58177	0.03807
PLD1	0.634202	0.028639	C6orf59	0.594944	0.048149	ARMCX6	-0.48813	0.03880
SNORD38A	0.609162	0.031006	ANKRD20A5	0.716681	0.048149	GUCY1A3	-0.81410	0.03992
TXNDC11	0.533694	0.032626	CD3D	0.688385	0.048459	PECAM1	-0.62322	0.04015
RPS15A	0.759417	0.032626	RPS18	0.688500	0.048995	DOCK8	-0.49090	0.04073
SP140	0.518082	0.032626	TRIM46	0.622758	0.049168	CLTC	-0.48625	0.04214
SNHG1	0.591483	0.033389	ZFP36L2	-0.714002	0.006793	CR609936	-0.71788	0.04214
C12orf27	0.780144	0.033869	TSC22D1	-1.216208	0.008222	POTEM	-0.64835	0.04214
RPS15A	0.719984	0.034611	DYRK1A	-0.803360	0.009573	PILRA	-0.73835	0.04225
NPCR	0.906927	0.035046	RNF44	-0.595669	0.011724	SDCBP	-0.76269	0.04244
NT5C	0.423967	0.035208	PIK3CB	-0.727278	0.012077	CHMP6	-0.52607	0.04296
BQ068637	0.479684	0.037108	NPEPPS	-0.632031	0.016906	EIF4G2	-0.64303	0.04296
AK021556	0.501986	0.037185	MSRA	-0.668972	0.020617	WLS	-0.78067	0.04318
CR604709	0.674991	0.037185	CA2	-0.949080	0.021554	CCM2	-0.56719	0.04348
LUC7L	0.483210	0.037185	FAR2	-0.731739	0.021554	COMT	-0.56333	0.04348
PEX16	0.492282	0.037185	TSC22D1	-0.784290	0.023820	ADCY7	-0.55099	0.04422
EBLN2	0.579082	0.037662	MAEA	-0.777216	0.024867	NRBF2	-0.56733	0.04422
STAT2	0.469548	0.038068	NCKAP5	-0.548263	0.025257	MEGF9	-0.60064	0.04450

SYMBOL	logFC	adj.P.Val	SYMBOL	logFC	adj.P.Val	SYMBOL	logFC	adj.P.Val
TNPO1	0.574401	0.038068	LAMP2	-0.645710	0.025542	AKAP13	-0.56658	0.04465
TTC5	0.487220	0.038796	BM984396	-0.867864	0.025542	SPARC	-0.98071	0.04465
REC9	0.521711	0.044260	MANSC1	-0.914461	0.026387	CBL	-0.52060	0.04496
PAQR9	0.514391	0.045133	BAMBI	-0.923699	0.027381	JMJD1C	-0.60674	0.04496
GVIN2	0.507071	0.046005	CA2	-0.952973	0.028413	MAP7D1	-0.43599	0.04496
LOC100188950	0.499751	0.046878	FAR2	-0.710137	0.028413	SAP130	-0.50321	0.04496
RPL16	0.492431	0.047751	LIMS1	-0.588895	0.028413	TRIM8	-0.43388	0.04496
UAP1L2	0.485111	0.048623	SLC39A8	-1.015622	0.028413	ZZEF1	-0.44339	0.04496
BX118466	0.477791	0.049496	MYADM	-0.728114	0.028639	LOC729451	-0.52772	0.04550
BX120212	0.470471	0.050369	MAEA	-0.598161	0.030652	RFX1	-0.48595	0.04609
CDKN2D	0.463151	0.051241	ABCB1	-0.659164	0.031694	SETD1B	-0.45472	0.04631
CD73	0.455831	0.052114	JAK1	-0.615790	0.032293	STXBP5	-0.46855	0.04654
SCARNA22	0.448511	0.052986	TSC22D1	-0.867901	0.032293	ITGB5	-0.61623	0.04815
HBD	0.441191	0.053859	ARF3	-0.668959	0.032365	CTR9	-0.50786	0.04816

Suppl.. Table 1b: List of genes differentially regulated at baseline between non-CC vs Control sorted by Iadj p value in ascending order. Adjusted P value was generated using moderated t-test in limma package

SYMBOL	logFC	adj.P.Val	SYMBOL	logFC	adj.P.Val	SYMBOL	logFC	adj.P.Val
RSAD2	1.90127	0.00226	TLR10	0.70488	0.03789	ERBB2	-1.11937	0.01917
IFIT3	1.46837	0.00794	HERC6	0.68978	0.03805	NCOA6	-0.63678	0.02234
IFIT1	1.46281	0.01152	STAP1	0.89478	0.03835	ADCY9	-0.62406	0.02413
LAMR1P15	0.88793	0.01635	FAM159A	0.79933	0.03862	IL1R1	-0.76878	0.02449
IFI6	1.14569	0.01834	LOC731779	0.68614	0.03862	LOC100124692	-0.83764	0.02449
BOLA2B	0.69050	0.01834	RPSA	0.66409	0.03862	LOC100132287	-1.03455	0.02583
BOLA2B	0.71919	0.01917	B4GALT3	0.63239	0.04033	ACACB	-0.96456	0.02588
FAM89B	0.65912	0.02010	RPL13	0.76882	0.04087	LOC100133161	-1.29932	0.02588
FCGR1C	1.10095	0.02234	RPSA	0.76790	0.04087	WHSC1L1	-0.65414	0.02743
TNFAIP6	1.34524	0.02290	IFI35	0.66732	0.04087	STT3A	-0.48734	0.02960
UBE2L6	0.64387	0.02439	RAB34	0.65498	0.04087	PDLIM5	-0.76265	0.03082
LY6E	0.97916	0.02449	C10orf32-AS3MT	0.55173	0.04110	FLJ45340	-1.65725	0.03113
LAMR1P15	0.75035	0.02449	TRAT1	0.88441	0.04112	AUTS2	-0.73320	0.03180
RTP4	0.73815	0.02449	C1orf228	0.74727	0.04172	AMACR	-0.76754	0.03209
PRELID1	0.57435	0.02449	BOLA2B	0.60330	0.04210	EPHB4	-0.94710	0.03339
OAS3	1.22267	0.02539	RPS2P32	0.57639	0.04262	PLGLB1	-0.98781	0.03504
OAS1	1.03780	0.02569	SNRPB2	0.50295	0.04262	MPZL1	-0.69276	0.03602
TNFSF13B	0.72026	0.02743	BATF2	1.02814	0.04350	SPEN	-0.54283	0.03615
RPSA	0.70948	0.02834	SOCS1	0.69775	0.04350	TCEAL4	-0.62673	0.03615
LAMR1P15	0.72268	0.02875	MICA	0.63059	0.04394	NDUFS2	-0.56323	0.04172
BTLA	0.99368	0.02942	NOL10	0.57739	0.04418	RNF145	-0.85489	0.04172
OAS1	0.98580	0.02954	CMPK2	0.90787	0.04441	INSIG1	-0.94513	0.04262
TCRA	0.66642	0.03016	RPL13AP6	0.71895	0.04494	PALLD	-0.86860	0.04262
CARD14	0.84541	0.03209	BCAS4	0.73735	0.04560	SLC25A20	-0.70785	0.04262
IFI44	1.25719	0.03570	RPL10	0.61663	0.04671	CDH2	-1.45377	0.04350
BTLA	0.76094	0.03570	ZNF234	0.57576	0.04941	DOK4	-0.57622	0.04350
LOC100505502	0.71961	0.03602	MINK1	0.49447	0.04941	PPP1R3B	-0.81660	0.04350
RPSA	0.70466	0.03602	C12orf57	0.73102	0.04981	ACOX1	-0.71302	0.04358
DKFZp666K117	0.77376	0.03615	PRKCDBP	0.58703	0.04981	SPAG9	-0.73466	0.04392
RPLP0	0.65630	0.03615	DKFZP586I1420	0.51216	0.04981	ABLIM3	-0.79763	0.04560
RPL14	0.83908	0.03625	PPP1R12B	-0.86079	0.01134	EPDR1	-0.72040	0.04941
TRIM22	0.76507	0.03704	CYP4F3	-1.00366	0.01373	NDRG2	-1.00816	0.04967

Suppl.. Table 1c: Reactome pathways enrichment analysis showing pathways up-regulated at baseline CC vs control sorted by P value in decending order using CAMERA package in limma.

Reactome Pathway	NGenes	Direction	PValue	FDR
REACTOME_PEPTIDE_CHAIN_ELONGATION	74	Up	6.00E-09	3.23E-06
REACTOME_SRP_DEPENDENT_COTRANSLATIONAL_PROTEIN_TARGETING_TO_MEMBRANE	84	Up	9.62E-09	3.23E-06
REACTOME_NONSENSE_MEDIATED_DECAY_ENHANCED_BY_THE_EXON_JUNCTION_COMPLEX	83	Up	5.84E-08	1.31E-05
REACTOME_INTERFERON_ALPHA_BETA_SIGNALING	43	Up	1.92E-07	2.57E-05
REACTOME_3_UTR_MEDIATED_TRANSLATIONAL_REGULATION	85	Up	2.34E-07	2.61E-05
REACTOME_INFLUENZA_VIRAL_RNA_TRANSCRIPTION_AND_REPLICATION	86	Up	4.12E-07	3.48E-05
REACTOME_TRANSLATION	101	Up	1.11E-06	8.26E-05
REACTOME_FORMATION_OF_THE_TERNARY_COMPLEX_AND_SUBSEQUENTLY_THE_43S_COMPLEX	33	Up	3.53E-05	0.00155229
REACTOME_INFLUENZA_LIFE_CYCLE	102	Up	3.70E-05	0.00155229
REACTOME_METABOLISM_OF_MRNA	130	Up	6.00E-05	0.00214282
REACTOME_METABOLISM_OF_RNA	153	Up	0.000238	0.00618696
REACTOME_INTERFERON_SIGNALING	106	Up	0.00025817	0.00618696
REACTOME_NEGATIVE_REGULATORS_OF_RIG_I_MDA5_SIGNALING	25	Up	0.00066592	0.01441385
REACTOME_ACTIVATION_OF_THE_MRNA_UPON_BINDING_OF_THE_CAP_BINDING_COMPLEX_AND_EIFS_AND_SUBSEQUENT_BINDING_TO_43S	39	Up	0.00080429	0.01583272
REACTOME_RIG_I_MDA5_MEDIATED_INDUCTION_OF_IFN_ALPHA_BETA_PATHWAYS	46	Up	0.00588061	0.05889391
REACTOME_INTERFERON_GAMMA_SIGNALING	49	Up	0.0185523	0.11526475
REACTOME_ACTIVATION_OF_IRF3_IRF7_MEDIATED_BY_TBK1_IKK_EPSILON	10	Up	0.0189167	0.11645052
REACTOME_METABOLISM_OF_PROTEINS	225	Up	0.02247905	0.13116036
REACTOME_REGULATION_OF_IFNA_SIGNALING	9	Up	0.03004098	0.15715437
REACTOME_RNA_POL_III_TRANSCRIPTION_INITIATION_FROM_TYPE_3_PROMOTER	13	Up	0.04351437	0.19728473
REACTOME_RNA_POL_III_CHAIN_ELONGATION	10	Up	0.05411748	0.22695519
REACTOME_ANTIVIRAL_MECHANISM_BY_IFN_STIMULATED_GENES	45	Up	0.05751696	0.23619989

Suppl. Table 1d. Reactome analysis showing pathways up-regulated at baseline non-CC vs Control sorted by P value in decending order using CAMERA package in limma.

Reactome Pathway	NGenes	Direction	PValue	FDR
INTERFERON_ALPHA_BETA_SIGNALING	43	Up	2.91E-14	1.95E-11
PEPTIDE_CHAIN_ELONGATION	74	Up	1.45E-12	4.87E-10
SRP_DEPENDENT_COTRANSLATIONAL_PROTEIN_TARGETING_TO_MEMBRANE	84	Up	4.52E-11	1.01E-08
3_UTR_MEDIATED_TRANSLATIONAL_REGULATION	85	Up	6.75E-11	1.13E-08
NONSENSE_MEDIATED_DECAY_ENHANCED_BY_THE_EXON_JUNCTION_COMPLEX	83	Up	5.55E-10	7.45E-08
INFLUENZA_VIRAL_RNA_TRANSCRIPTION_AND_REPLICATION	86	Up	8.93E-10	9.99E-08
TRANSLATION	101	Up	1.22E-09	1.17E-07
INTERFERON_SIGNALING	106	Up	1.33E-08	1.12E-06
FORMATION_OF_THE_TERNARY_COMPLEX_AND_SUBSEQUENTLY_THE_43S_COMPLEX	33	Up	9.91E-08	7.39E-06
INFLUENZA_LIFE_CYCLE	102	Up	1.67E-07	1.12E-05
METABOLISM_OF_MRNA	130	Up	2.31E-06	0.00012901
INTERFERON_GAMMA_SIGNALING	49	Up	4.25E-06	0.00021913
ACTIVATION_OF_THE_MRNA_UPON_BINDING_OF_THE_CAP_BINDING_COMPLEX_AND_EIFS_AND_SUBSEQUENT_BINDING_TO_43S	39	Up	1.18E-05	0.0005296
METABOLISM_OF_RNA	153	Up	1.94E-05	0.00078746
NEGATIVE_REGULATORS_OF_RIG_I_MDA5_SIGNALING	25	Up	0.00011677	0.00340667
METABOLISM_OF_PROTEINS	225	Up	0.00084629	0.01622455
CYTOKINE_SIGNALING_IN_IMMUNE_SYSTEM	178	Up	0.00105004	0.01795018
RIG_I_MDA5_MEDIATED_INDUCTION_OF_IFN_ALPHA_BETA_PATHWAYS	46	Up	0.0016778	0.02447399
REGULATION_OF_IFNA_SIGNALING	9	Up	0.00291336	0.03688429
ACTIVATION_OF_IRF3_IRF7_MEDIATED_BY_TBK1_IKK_EPSILON	10	Up	0.00384138	0.04114558
PURINE_SALVAGE	7	Up	0.00410259	0.04301311
ANTIVIRAL_MECHANISM_BY_IFN_STIMULATED_GENES	45	Up	0.00636153	0.05928595
TRAF6_MEDIATED_IRF7_ACTIVATION	15	Up	0.01966772	0.12450035
PURINE_METABOLISM	15	Up	0.02229255	0.13014379
CROSS_PRESENTATION_OF_SOLUBLE_EXOGENOUS_ANTIGENS_ENDOSOMES	20	Up	0.03104863	0.16404433
ER_PHAGOSOME_PATHWAY	27	Up	0.03262934	0.16713195
VIF_MEDIATED_DEGRADATION_OF_APOBEC3G	24	Up	0.03468838	0.17370075
SYNTHESIS_OF_DNA	35	Up	0.03879261	0.18460881
RESPIRATORY_ELECTRON_TRANSPORT_ATP_SYNTHESIS_BY_CHEMIOSMOTIC_COUPLING_AND_HEAT_PRODUCTION_BY_UNCOUPLING_PROTEINS_	53	Up	0.04003249	0.18654028
FORMATION_OF_ATP_BY_CHEMIOSMOTIC_COUPLING	10	Up	0.04437004	0.19848196
PD1_SIGNALING	12	Up	0.04588142	0.20287086
TRANSLOCATION_OF_ZAP_70_TO_IMMUNOLOGICAL_SYNAPSE	11	Up	0.04595584	0.20287086
REGULATION_OF_MITOTIC_CELL_CYCLE	29	Up	0.04788884	0.20598341
ASSOCIATION_OF_TRIC_CCT_WITH_TARGET_PROTEINS_DURING_BIOSYNTHESIS	15	Up	0.05397504	0.21817623
TRAF3_DEPENDENT_IRF_ACTIVATION_PATHWAY	11	Up	0.05646431	0.22411791
S_PHASE	44	Up	0.05664959	0.22411791
CDT1_ASSOCIATION_WITH_THE_CDC6_ORC_ORIGIN_COMPLEX	23	Up	0.06012073	0.23073097
SCF_BETA_TRCP_MEDIATED_DEGRADATION_OF_EMI1	21	Up	0.06470483	0.24255276
SCFSKP2_MEDIATED_DEGRADATION_OF_P27_P21	21	Up	0.06665984	0.24712017
CLASS_I_MHC_MEDIATED_ANTIGEN_PROCESSING_PRESENTATION	125	Up	0.06756691	0.24910658

Suppl. Table 2a. Reactome pathways enrichment analysis showing overrepresented pathways in up-regulated genes in CC patients at 16 weeks Vs 4 weeks.

Reactome Pathway	NGenes	Direction	PValue	FDR
BIOLOGICAL_OXIDATIONS	80	Up	1.29E-08	8.66E-06
BILE_ACID_AND_BILE_SALT_METABOLISM	16	Up	2.44E-07	8.17E-05
COMPLEMENT_CASCADE	27	Up	1.17E-06	0.000262
LIPOPROTEIN_METABOLISM	23	Up	1.92E-06	0.00029875
PHASE_II_CONJUGATION	39	Up	2.23E-06	0.00029875
SYNTHESIS_OF_BILE_ACIDS_AND_BILE_SALTS_VIA_7ALPHA_HYDROXYCHOLESTEROL	11	Up	5.57E-06	0.00052821
SYNTHESIS_OF_BILE_ACIDS_AND_BILE_SALTS	11	Up	5.57E-06	0.00052821
RESPONSE_TO_ELEVATED_PLATELET_CYTOSOLIC_CA2_	56	Up	6.44E-06	0.00052821
LIPID_DIGESTION_MOBILIZATION_AND_TRANSPORT	29	Up	7.08E-06	0.00052821
PHASE1_FUNCTIONALIZATION_OF_COMPOUNDS	42	Up	7.93E-06	0.00053235
CYTOCHROME_P450_ARRANGED_BY_SUBSTRATE_TYPE	25	Up	1.24E-05	0.00075931
METABOLISM_OF_AMINO_ACIDS_AND_DERIVATIVES	112	Up	1.66E-05	0.00085846
XENOBIOTICS	10	Up	2.33E-05	0.00111712
FORMATION_OF_FIBRIN_CLOT_CLOTTING_CASCADE	25	Up	4.92E-05	0.00199598
GLUTATHIONE_CONJUGATION	16	Up	5.06E-05	0.00199598
INITIAL_TRIGGERING_OF_COMPLEMENT	12	Up	6.84E-05	0.00255018
COMMON_PATHWAY	13	Up	7.34E-05	0.00259141
HDL_MEDIATED_LIPID_TRANSPORT	12	Up	8.30E-05	0.00278445
FATTY_ACID_TRIACYLGLYCEROL_AND_KETONE_BODY_METABOLISM	100	Up	8.93E-05	0.00285386
ADHERENS_JUNCTIONS_INTERACTIONS	8	Up	0.00011727	0.00354131
ALPHA_LINOLENIC_ACID_ALA_METABOLISM	6	Up	0.00014235	0.00397984
SYNTHESIS_OF_BILE_ACIDS_AND_BILE_SALTS_VIA_24_HYDROXYCHOLESTEROL	8	Up	0.00020101	0.00518768
CHYLOMICRON_MEDIATED_LIPID_TRANSPORT	13	Up	0.000281	0.00673407
METABOLISM_OF_LIPIDS_AND_LIPOPROTEINS	260	Up	0.00030376	0.00702841

Reactome Pathway	NGenes	Direction	PValue	FDR
CELL_CELL_JUNCTION_ORGANIZATION	19	Up	0.00100742	0.0193137
INTERFERON_ALPHA_BETA_SIGNALING	43	Up	0.00104696	0.01951425
RECYCLING_OF_BILE_ACIDS_AND_SALTS	7	Up	0.00121274	0.0218816
MITOCHONDRIAL_FATTY_ACID_BETA_OXIDATION	9	Up	0.00169218	0.02838625
ETHANOL_OXIDATION	9	Up	0.00184679	0.03022425
CREATION_OF_C4_AND_C2_ACTIVATORS	7	Up	0.00197676	0.03158111
GLUCURONIDATION	6	Up	0.00237959	0.0371326
PEROXISOMAL_LIPID_METABOLISM	13	Up	0.00290354	0.04204556
PLATELET_ACTIVATION_SIGNALING_AND_AGGREGATION	127	Up	0.00300773	0.04204556
BRANCHED_CHAIN_AMINO_ACID_CATABOLISM	14	Up	0.00357329	0.04785458
SULFUR_AMINO_ACID_METABOLISM	16	Up	0.00363723	0.04785458
GLUCOSE_METABOLISM	33	Up	0.00399502	0.05155113
REGULATION_OF_COMPLEMENT_CASCADE	12	Up	0.00491153	0.06103034
TRIGLYCERIDE_BIOSYNTHESIS	22	Up	0.0051702	0.06307647
COLLAGEN_FORMATION	16	Up	0.0061621	0.07383518
INTRINSIC_PATHWAY	12	Up	0.00660589	0.07776402
ENOS_ACTIVATION_AND_REGULATION	12	Up	0.00814438	0.09422204
CELL_JUNCTION_ORGANIZATION	29	Up	0.0087289	0.09632794
AMYLOIDS	36	Up	0.00875709	0.09632794
EXTRACELLULAR_MATRIX_ORGANIZATION	26	Up	0.01063838	0.11330716
PPARA_ACTIVATES_GENE_EXPRESSION	61	Up	0.01128632	0.11650949
TRYPTOPHAN_CATABOLISM	10	Up	0.0118743	0.12072207
SMOOTH_MUSCLE_CONTRACTION	15	Up	0.01213489	0.12152999
REGULATION_OF_INSULIN_LIKE_GROWTH_FACTOR_IGF_ACTIVITY_BY_INSULIN_LIKE_GROWTH_FACTOR_BINDING_PROTEINS_IGFBPS	8	Up	0.01322376	0.13048743
AMINO_ACID_SYNTHESIS_AND_INTERCONVERSION_TRANSAMINATION	10	Up	0.01387749	0.13495355
IL_6_SIGNALING	8	Up	0.01544535	0.147757

Suppl. Table 2b. Reactome pathways enrichment analysis showing overrepresented pathways in down-regulated genes in non-CC patients 16 weeks Vs 4 weeks.(i.e pathways enriched at 4 weeks)

Reactome Pathways	NGenes	Direction	PValue	FDR
GENERATION_OF_SECOND_MESSENGER_MOLECULES	20	Down	0.00051233	0.0087811
TCR_SIGNALING	33	Down	0.00071087	0.01124595
LATENT_INFECTION_OF_HOMO_SAPIENS_WITH_MYCOBACTERIUM_TUBERCULOSIS	16	Down	0.00072068	0.01124595
IL_2_SIGNALING	29	Down	0.00223589	0.03125585
RAP1_SIGNALLING	11	Down	0.00256071	0.03436478
SIGNALING_BY_ILS	71	Down	0.00355362	0.04499014
NEUROTRANSMITTER_RECEPTOR_BINDING_AND_DOWNSTREAM_TRANSMISSION_IN_THE_POSTSYNAPTIC_CELL	46	Down	0.00472685	0.05873553
IMMUNOREGULATORY_INTERACTIONS_BETWEEN_A_LYMPHOID_AND_A_NON_LYMPHOID_CELL	47	Down	0.00584806	0.06650934
G_PROTEIN_BETA_GAMMA_SIGNALLING	16	Down	0.00620893	0.06943652
ANTIGEN_ACTIVATES_B_CELL_RECEPTOR_LEADING_TO_GENERATION_OF_SECOND_MESSENGERS	23	Down	0.00683894	0.07522831
TRANSLOCATION_OF_ZAP_70_TO_IMMUNOLOGICAL_SYNAPSE	11	Down	0.00788383	0.08532342
G_ALPHA_Z_SIGNALLING_EVENTS	21	Down	0.00890465	0.09053064
CYTOKINE_SIGNALING_IN_IMMUNE_SYSTEM	178	Down	0.00977219	0.09786776
ADAPTIVE_IMMUNE_SYSTEM	297	Down	0.00994668	0.09815031
EFFECTS_OF_PIP2_HYDROLYSIS	11	Down	0.01128375	0.10816283
G_BETA_GAMMA_SIGNALLING_THROUGH_PLC_BETA	11	Down	0.01167478	0.11033486
REGULATION_OF_INSULIN_SECRETION_BY_GLUCAGON_LIKE_PEPTIDE1	15	Down	0.01197498	0.11160017
ACTIVATION_OF_KAINATE_RECEPTORS_UPON_GLUTAMATE_BINDING	13	Down	0.01380752	0.12565943
PD1_SIGNALING	12	Down	0.01385812	0.12565943
IL_3_5_AND_GM_CSF_SIGNALING	31	Down	0.01462108	0.13080991
PLC_BETA_MEDIATED_EVENTS	17	Down	0.01598717	0.1363263

Reactome Pathways	NGenes	Direction	PValue	FDR
PLATELET_HOMEOSTASIS	36	Down	0.0174837	0.14454835
GENERIC_TRANSCRIPTION_PATHWAY	143	Down	0.01873756	0.14967745
COSTIMULATION_BY_THE_CD28_FAMILY	41	Down	0.02081882	0.15915014
PROSTACYCLIN_SIGNALLING_THROUGH_PROSTACYCLIN_RECEPTOR	10	Down	0.02087215	0.15915014
G_BETA_GAMMA_SIGNALLING_THROUGH_PI3KGAMMA	14	Down	0.0215405	0.15922917
G_ALPHA_S_SIGNALLING_EVENTS	34	Down	0.02173652	0.15922917
SIGNALLING_TO_ERKS	21	Down	0.02204379	0.15922917
IL_RECEPTOR_SHC_SIGNALING	19	Down	0.02308837	0.16307679
PHOSPHORYLATION_OF_CD3_AND_TCR_ZETA_CHAINS	12	Down	0.02408386	0.16833613
INTERFERON_ALPHA_BETA_SIGNALING	43	Down	0.02479096	0.17079789
DOWNSTREAM_TCR_SIGNALING	21	Down	0.02494515	0.17079789
GASTRIN_CREB_SIGNALLING_PATHWAY_VIA_PKC_AND_MAPK	83	Down	0.02836109	0.1823814
GABA_B_RECEPTOR_ACTIVATION	15	Down	0.02853956	0.1823814
GABA_RECEPTOR_ACTIVATION	15	Down	0.02853956	0.1823814
OPIOID_SIGNALLING	35	Down	0.03165301	0.20036953
TRAF6_MEDIATED_IRF7_ACTIVATION_IN_TLR7_8_OR_9_SIGNALING	6	Down	0.03363306	0.21091387
PROLONGED_ERK_ACTIVATION_EVENTS	12	Down	0.03487347	0.21666758
RESOLUTION_OF_AP_SITES_VIA_THE_MULTIPLE_NUCLEOTIDE_PATCH_REPLACEMENT_PATHWAY	6	Down	0.03606824	0.22173779
G_PROTEIN_ACTIVATION	12	Down	0.03759798	0.22525218
TRANSPORT_OF_MATURE_TRANSCRIPT_TO_CYTOPLASM	28	Down	0.03845741	0.22681651
ADP_SIGNALLING_THROUGH_P2RY12	11	Down	0.03853515	0.22681651
SIGNALING_BY_GPCR	226	Down	0.03912425	0.22828149
GPCR_DOWNSTREAM_SIGNALING	183	Down	0.04067097	0.23324972
INHIBITION_OF_INSULIN_SECRETION_BY_ADRENALINE_NORADRENALINE	11	Down	0.04141621	0.2338961
PROSTANOID_LIGAND_RECEPTORS	3	Down	0.04250639	0.23768157
G_ALPHA_Q_SIGNALLING_EVENTS	67	Down	0.04342482	0.24022094
INHIBITION_OF_VOLTAGE_GATED_CA2_CHANNELS_VIA_GBETA_GAMMA_SUBUNITS	10	Down	0.04367654	0.24022094

Reactome Pathways	NGenes	Direction	PValue	FDR
SIGNALLING_TO_P38_VIA_RIT_AND_RIN	9	Down	0.04546551	0.24529688
TRANSPORT_OF_RIBONUCLEOPROTEINS_INTO_THE_HOST_NUCLEUS	14	Down	0.04606171	0.24529688
